# Angelica gigas Nakai and Decursin Downregulate Myc Expression to Promote Cell Death in B-cell Lymphoma

**DOI:** 10.1038/s41598-018-28619-z

**Published:** 2018-07-12

**Authors:** Eungyoung Kim, Jehyun Nam, Woochul Chang, Ismayil S. Zulfugarov, Zhanna M. Okhlopkova, Daniil Olennikov, Nadezhda K. Chirikova, Sang-Woo Kim

**Affiliations:** 1 Department of Integrated Biological Science, Pusan National University, Pusan, 46241 Republic of Korea; 2 Department of Biology Education, Pusan National University, Pusan, 46241 Republic of Korea; 3 Department of Molecular Biology, Pusan National University, Pusan, 46241 Republic of Korea; 4 Department of Biology, North-Eastern Federal University, 58 Belinsky Str., Yakutsk, 677027 Russia; 5 Institute of Molecular Biology and Biotechnology, Azerbaijan National Academy of Sciences, Matbuat Avenue 2a, Baku, AZ 1073 Azerbaijan; 6 Institute of General and Experimental Biology, Sakh’yanovoy Str. 6, Ulan-Ude, Russia; 7 Department of Biological Sciences, Pusan National University, Pusan, 46241 Republic of Korea

## Abstract

*Angelica gigas* Nakai (AGN) is an oriental traditional medicine to treat anemia, dysmenorrhea, and migraine. However, its anti-lymphoma effect is yet to be tested. Here, we demonstrated that AGN and its major component decursin target *Myc* to suppress lymphomagenesis *in vitro* and *in vivo*. AGN inhibited cell viability in multiple B lymphoma cells, while sparing normal splenocytes and bone marrow cells. Increased cleaved PARP level and caspase 3/7 activity and the repression of survival-promoting AKT/mTOR and MAPK pathways downstream of BCR, were responsible for the pro-apoptotic effects of AGN. We found that *Myc*, a prominent downstream target of these signaling pathways, contributes to AGN-induced cell death. Moreover, co-treatment with AGN and a Myc inhibitor, JQ1 or 10058-F4 yielded synergistic cytotoxic activities against cancer cells with markedly reduced *Myc* expression. AGN downregulated *Myc* expression and suppressed tumorigenesis in Eμ-myc transgenic mice. The proapoptotic activities of AGN were recapitulated by decursin, indicating that the anti-tumor effect of AGN was mainly caused by decursin. These findings suggest that AGN and decursin possess potent anti-lymphoma activity, and combination therapies with AGN/decursin and a Myc inhibitor to target Myc more efficiently could be a valuable avenue to explore in the treatment of B-cell lymphoma.

## Introduction

Lymphoma is the most common form of blood cancer that involves B-lymphocytes, T-lymphocytes, and natural killer cells. It is divided into Hodgkin’s lymphoma and non-Hodgkin’s lymphoma (NHL). NHL mainly involves B cells and B-cell lymphoma accounts for 85% of all lymphoma cases. B-cell lymphoma includes DLBCL, Burkitt’s lymphoma, follicular lymphoma, and mantle cell lymphoma^1,2^. DLBCL is the most frequently diagnosed NHL, and accounts for more than 41% of NHL^3,4^. Despite recent advances in treatment strategies, DLBCL remains a serious concern^5,6^. Therefore, there is a need to develop novel improved therapeutic alternatives to treat DLBCL more effectively.

Oriental herbs have long been used in Asian countries, such as China, Japan, and Korea, to treat various diseases. Herbal therapies have recently attracted attention due to their safety and therapeutic effects. AGN is one of the most commonly used herbs and it has been shown to exert anti-inflammatory, anti-oxidant, and anti-cancer effects. Decursin, one of the major components of AGN, has anti-proliferative and apoptotic activities by regulating various cell growth signaling pathways in several types of human cancers^7^. However, anti-tumorigenic effects of AGN and decursin have not been tested in DLBCL.

The pathogenesis of DLBCL is associated with various growth-promoting signals. One of the critical targets of these pathways is the *c-Myc* (hereafter Myc) proto-oncogene. Although the *Myc* proto-oncogene is tightly regulated in normal cells, it is abnormally regulated in tumor cells at the transcriptional and post-transcriptional levels. *Myc* gene dysregulation has been observed in lymphoid neoplasia^8–12^. Molecular mechanisms by which *Myc* contributes to tumorigenesis are mostly related to *Myc* overexpression. The translocation of *Myc* to the immunoglobulin (Ig) locus, leading to its overexpression, occurs in most Burkitt’s lymphomas. The rearrangement and amplification of *Myc* are also frequently identified in DLBCL^2,13^. Eμ-myc transgenic mouse model is commonly used to simulate Myc-induced lymphoma; in these transgenic mice, the *Myc* gene is introduced in the lymphoid-specific Ig heavy chain (IgH) locus. Approximately 90% of Eμ-myc mice invariably develop B-cell lymphomas during the first five months^11,14–16^.

Most growth factors bind to cell-surface receptors and then induce the auto-phosphorylation of receptor tyrosine kinases, which activate downstream signaling proteins and regulate gene transcription. B cell receptor (BCR) is one of the critical signaling molecules for the survival and differentiation of both normal and malignant B cells. It is an Ig molecule that forms a type I transmembrane protein on the surface of B cells. It transduces activated signals in the B cell following its recognition of a specific antigen^17,18^. The binding of ligands or antigens to BCR leads to the phosphorylation of downstream proteins, inducing the activation of proteins with phosphotyrosine-binding SH2 domains, such as phosphatidylinositol 3-kinase (PI3K) and Bruton’s tyrosine kinase (BTK). PI3K phosphorylation induces the formation of PIP3, which in turn activates AKT. Activated AKT triggers the phosphorylation/activation of various substrates involved in the regulation of cell survival and cellular growth. BTK, another critical component of BCR signaling, is involved in B cell development. BTK phosphorylates phospholipase C, which hydrolyzes phosphatidylinositol 4,5-bisphosphoate (PIP2) into inositol triphosphate (IP3) and diacylglycerol (DAG). These two secondary messengers regulate gene expression by activating proteins involved in NF-κB and MAPK pathways. NF-κB is a transcription factor that promotes inflammation, B cell survival, proliferation, and differentiation. MAPK also facilitates cell proliferation. Abnormalities in BCR signaling are associated with chronic lymphocytic leukemia and B-cell lymphomas^19–21^. Indeed, numerous anti-cancer therapies target BCR and downstream proteins to treat these types of malignancies^22^.

In this study, we investigated the anti-lymphoma effects of AGN and its major compound decursin *in vitro* and *in vivo*. Our study suggested that AGN and decursin might be effective in inducing cytotoxicity by targeting Myc, a critical downstream molecule of BCR signaling, and their combination with Myc inhibitors could provide synergistic chemotherapeutic effects for the treatment of B-cell lymphomas.

## Results

### AGN induces apoptosis in DLBCL cell lines

**Figure 1:**
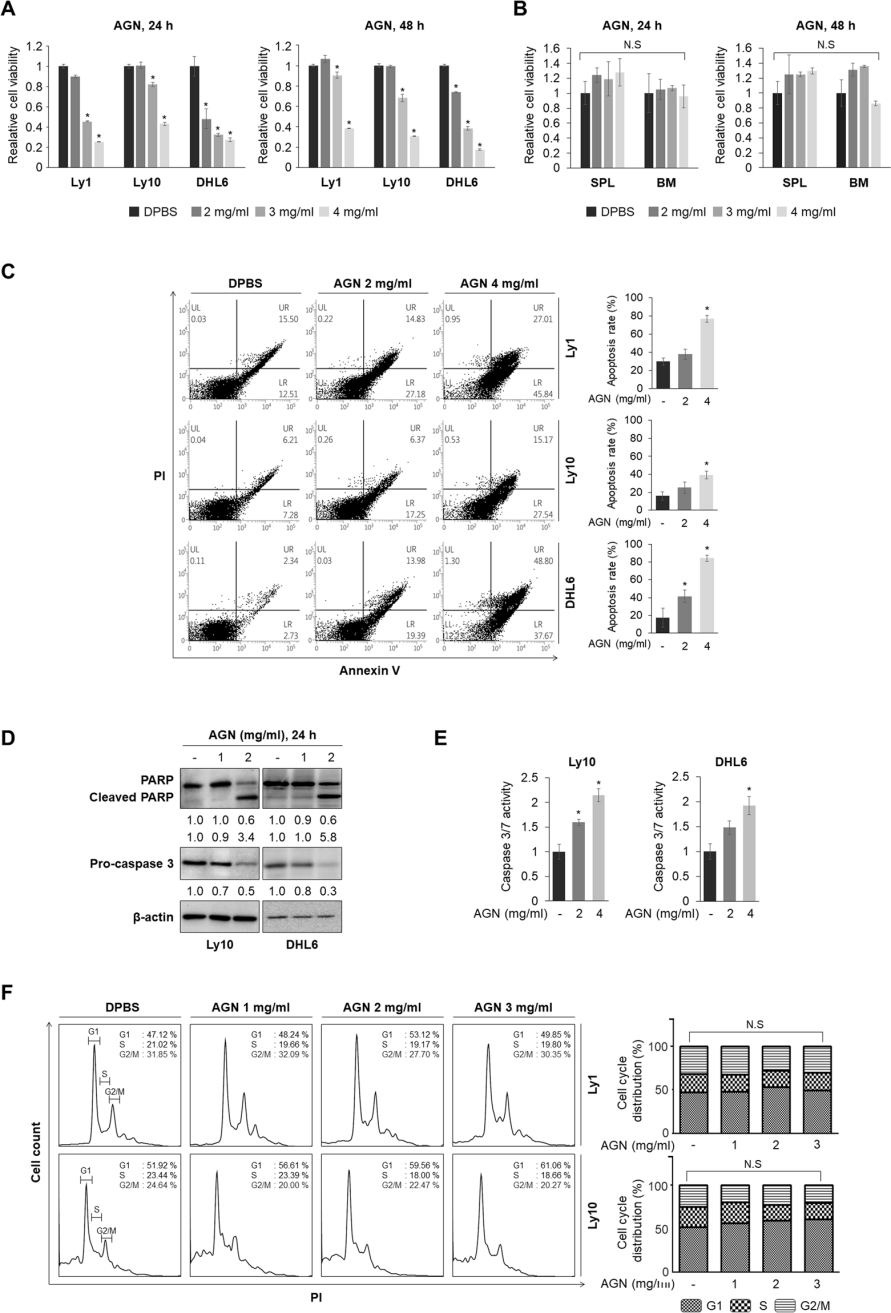
AGN causes cell death by activating caspase 3/7. (**A**) Three DLBCL cell lines (Ly1, Ly10, and DHL6) and (**B**) splenocytes and bone marrow cells from wild-type mice were exposed to AGN (0, 2, 3, or 4 mg/ml) for 24 and 48 h and MTS assays were performed to measure cell viability. The statistical significance was calculated using a two-tailed Student’s *t* test (*p < 0.05). n.s: not significant, SPL: splenocyte, BM: bone marrow. (**C**) AGN treatment for 24 h increases apoptosis in a dose-dependent manner in Ly1, Ly10, and DHL6 cells when analyzed by flow cytometry after staining with Annexin V-FITC and PI. A two-tailed Student’s *t* test is used to calculate statistical significance (*p < 0.05). (**D**) Cells were treated with AGN (0, 1, or 2 mg/ml) for 24 h. Whole cell lysates were subjected to western blot assays with antibodies against PARP, cleaved PARP, pro-caspase 3, and β-actin (internal standard). (**E**) Caspase 3/7 activities were measured by ELISA-based bioluminescence assays following treatment with AGN (0, 2, or 4 mg/ml). (**F**) Treatment with AGN (0, 1, 2, or 3 mg/ml) for 24 h in DLBCL cell lines did not have any effect on cell cycle distribution as analyzed by flow cytometry after staining with PI. Representative data from at least three independent experiments are shown.

AGN exerts anti-inflammatory, anti-angiogenesis, and anti-tumor effects in breast, lung, and prostate cancer^7^. To investigate whether AGN has anti-lymphoma activities, Ly1, Ly10, and DHL6 DLBDL cell lines were exposed to increasing concentrations of AGN for 24 and 48 h. Cell viability analysis indicated that AGN induces cytotoxicity in a dose-dependent manner in Ly1, Ly10, and DHL6 cells with negligible effect on normal splenocytes and bone marrow cells isolated from wild-type mice (Fig. 1A,B). To further characterize the cytotoxic effect of AGN on DLBCL cells, the cells were treated with AGN, followed by the measurement of apoptotic rates. FACS analysis after staining with Annexin V and PI revealed that exposure to AGN considerably increased the proportion of apoptotic population in all three DLBCL cell lines (Fig. 1C). Consistent with these findings, cleaved PARP level and caspase 3/7 activity, which are markers of apoptosis, increased in a dose-dependent manner (Fig. 1D,E and Fig. S1), indicating that AGN drives apoptosis. Moreover, flow cytometric analysis of the cell cycle revealed that distributions of cells in G_0_/G_1_, S, and G_2_/M cell cycle phases were not perturbed by treatment with AGN (Fig. 1F). We were unable to obtain the cell cycle profile at the concentration of 4 mg/ml of AGN because most of the cells were killed at this concentration. These results indicate that AGN treatment inhibits cell survival but not cell cycle progression in DLBCL cells.

### AGN downregulates Myc expression by inhibiting PI3K/AKT and MAPK pathways

**Figure 2:**
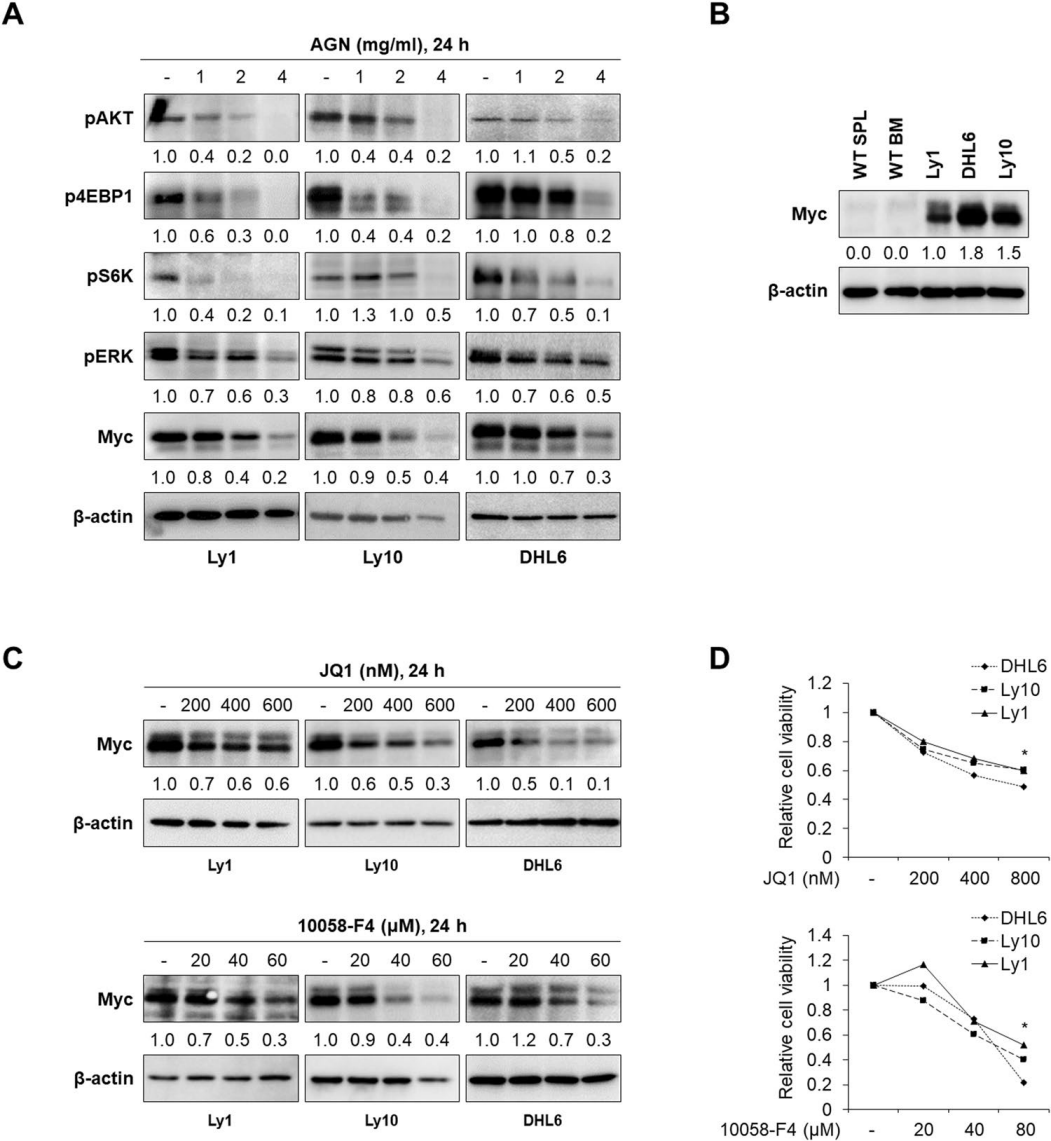
AGN targets Myc by suppressing PI3K/AKT and MAPK pathways. (**A**) Ly1, Ly10 and DHL6 cells were treated with vehicle or AGN (0, 1, 2, or 4 mg/ml) for 24 h. Myc protein levels and levels of pAKT, pS6K, p4EBP1, and pERK were detected by western blot assays. β-actin was used as a loading control. (**B**) Myc protein levels in three DLBCL cell lines and splenocytes and bone marrow cells from wild-type mice were determined by western blotting. Myc levels were increased in cancer cells compared with normal cells. (**C**) Myc protein levels were analyzed by western blot assays in DLBCL cell lines treated with JQ1 (0, 200, 400, or 600 nM) or 10058-F4 (0, 20, 40, or 60 μM). Representative blots from three separate experiments are shown. (**D**) Ly1, Ly10, and DHL6 cells were treated with increasing concentrations of JQ1 (0, 200, 400, or 800 nM) for 48 h or 10058-F4 (0, 20, 40, or 80 μM) for 24 h, and cell viability was determined by MTS assays. The statistical significance was calculated using a two-tailed Student’s *t* test (*p < 0.05).

Apoptotic and cell survival pathways are tightly regulated^17,23–25^, and BCR signaling has been shown to be critical for the survival of malignant B cells as well as normal B cells^17^. We hypothesized that AGN exerts cytotoxicity by inhibiting PI3K/AKT/mTOR and MAPK signaling, downstream pathways of BCR, the dysregulation of which has been shown to contribute to the formation of various human cancers, including DLBCL^26^. To test our hypothesis, DLBCL cells were exposed to AGN, followed by the western blot analysis of phosphorylated forms of AKT, S6K, 4EBP1, and ERK. As shown in Fig. 2A, the phosphorylation of AKT, S6K, and 4EBP1 was efficiently inhibited by AGN (Fig. S2). It also significantly attenuated the expression of Myc, a transcription factor known to be a downstream target of PI3K/AKT/mTOR and MAPK pathways. This is intriguing because the dysregulation of Myc expression by translocation or by other means has been shown to be directly associated with the aggressiveness of B-cell malignancies and the poor survival of these patients^9,10,12^. Consistent with the potential role of Myc in tumorigenesis, DLBCL cell lines displayed increased Myc expression compared with splenocytes and bone marrow cells from wild type mice (Fig. 2B and Fig. S3). To directly test whether Myc is vital for the survival of DLBCL cells in this setting, we treated these cells with two different types of Myc inhibitors^27,28^, JQ1 (a BET bromodomain inhibitor) and 10058-F4 (a Myc-Max dimerization inhibitor), which led to decreased Myc levels and cell viability^29,30^ (Fig. 2C,D and Fig. S4). This result suggests that AGN exerts anti-lymphoma effects largely by inhibiting Myc expression. These data collectively show that AGN suppresses Myc levels and cell viability by downmodulating PI3K/AKT/mTOR and MAPK signaling pathways.

### Combination treatment with AGN and a Myc inhibitor synergistically downregulates DLBCL cell viability

**Figure 3:**
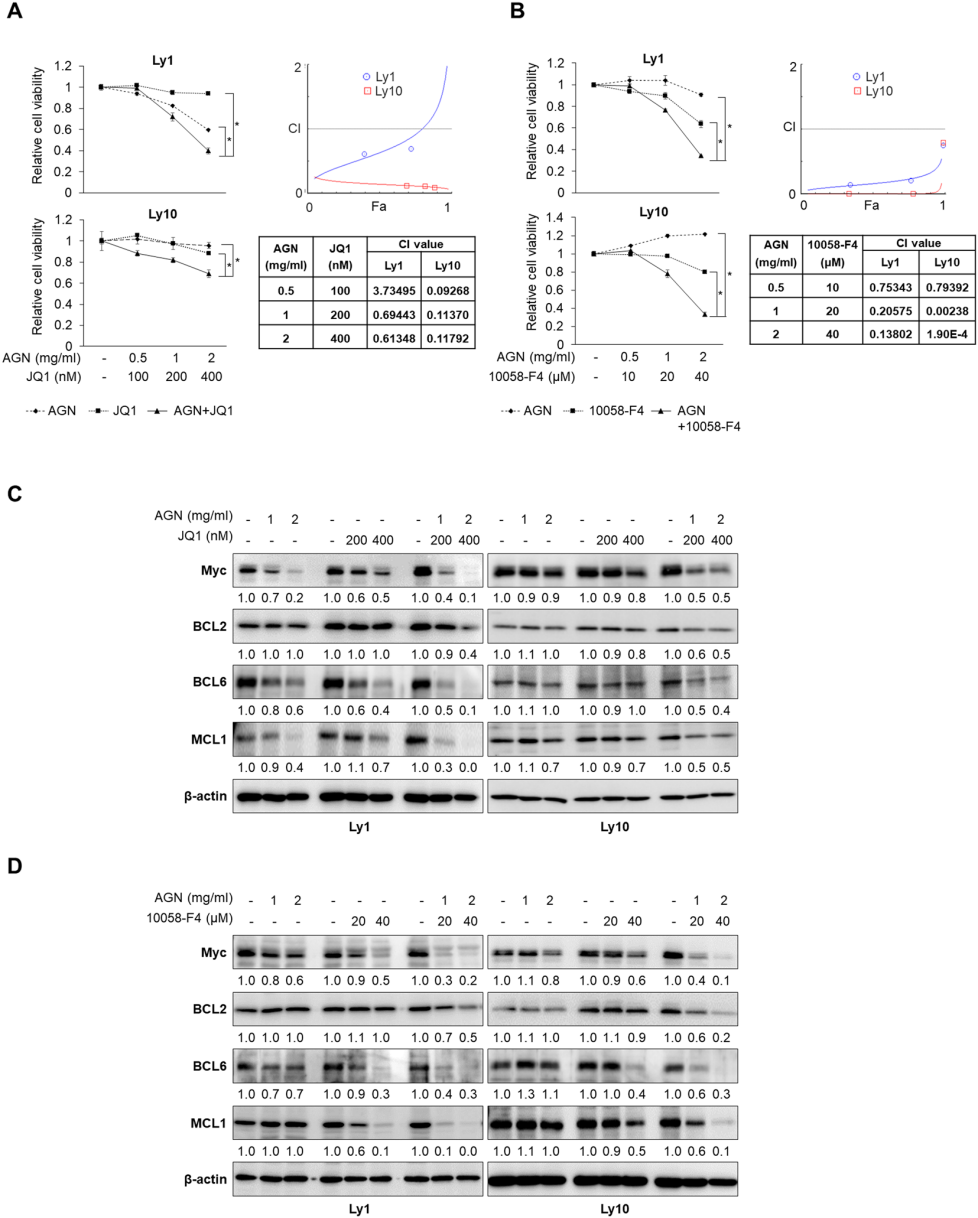
Combination therapy is more effective than monotherapy. (**A**) Two DLBCL cell lines (Ly1 and Ly10) were exposed to AGN (0, 1, or 2 mg/ml) and/or JQ1 (0, 200, or 400 nM) for 48 h, and MTS assays were performed to measure cell viability. Co-treatment with AGN and JQ1 markedly suppressed cell proliferation compared to treatment with AGN or JQ1 alone in DLBCL cell lines (Left panel). The statistical significance was calculated using a two-tailed Student’s *t* test (*p < 0.05). CI values were calculated using the CompuSyn software to quantify potential drug synergism (Right panel). (**B**) Ly1 and Ly10 cells were co-treated with AGN (0, 0.5, 1, or 2 mg/ml) and 10058-F4 (0, 10, 20, or 40 μM) or either agent alone for 48 h, and cell viability was determined by MTS assays (Left panel). The statistical significance was calculated using a two-tailed Student’s *t* test (*p < 0.05). Probable drug synergism was quantified using the Compusyn software (Right panel). (**C**) Cells were treated with AGN (0, 1, or 2 mg/ml) and/or JQ1 (0, 200, or 400 nM) for 48 h. Cell lysates were subjected to western blot assays with antibodies against Myc and anti-apoptotic proteins, BCL2, MCL1, and BCL6. β-actin was used as a loading control. (**D**) Western blot analysis was carried out to determine expression of Myc and anti-apoptotic proteins in Ly1 and Ly10 cells following incubation with AGN (0, 1, or 2 mg/ml) and/or 10058-F4 (0, 20, or 40 μM) for 48 h.

Because AGN does not completely abolish Myc expression, we hypothesized that targeting Myc with AGN along with a Myc inhibitor would synergistically inhibit Myc expression and cell survival. To this end, we exposed DLBCL cells with a combination of AGN and JQ1 for 48 h and measured cell survival rates by the MTS assay. Our data suggest that cell viability was inhibited more effectively by the combination than by AGN or JQ1 alone (Fig. 3A). Similarly, 10058-F4 significantly decreased the viability of Ly1 and Ly10 cells when combined with AGN (Fig. 3B). To quantitatively determine probable drug synergism, CI values were calculated by the CompuSyn software. Most data points indicated strong synergism when AGN is combined with either JQ1 or 10058-F4. BCL2 and its functionally redundant family member MCL1 are frequently overexpressed to cause drug resistance in DLBCL^6,31^. Western blot assays showed that AGN co-treatment with JQ1 or 10058-F4 markedly decreased the expression of BCL6, MCL1, and BCL2 (Fig. 3C,D, Fig. S5, and Fig. S6). These data highlight that the attenuation of the expression of BCL6 and pro-survival BCL2 members in addition to Myc expression contributes to the synergistic anti-cancer effects of combinations of AGN and Myc inhibitors, which may improve the outcomes of DLBCL treatment.

### Decursin reproduces cytotoxic activities of AGN

**Figure 4:**
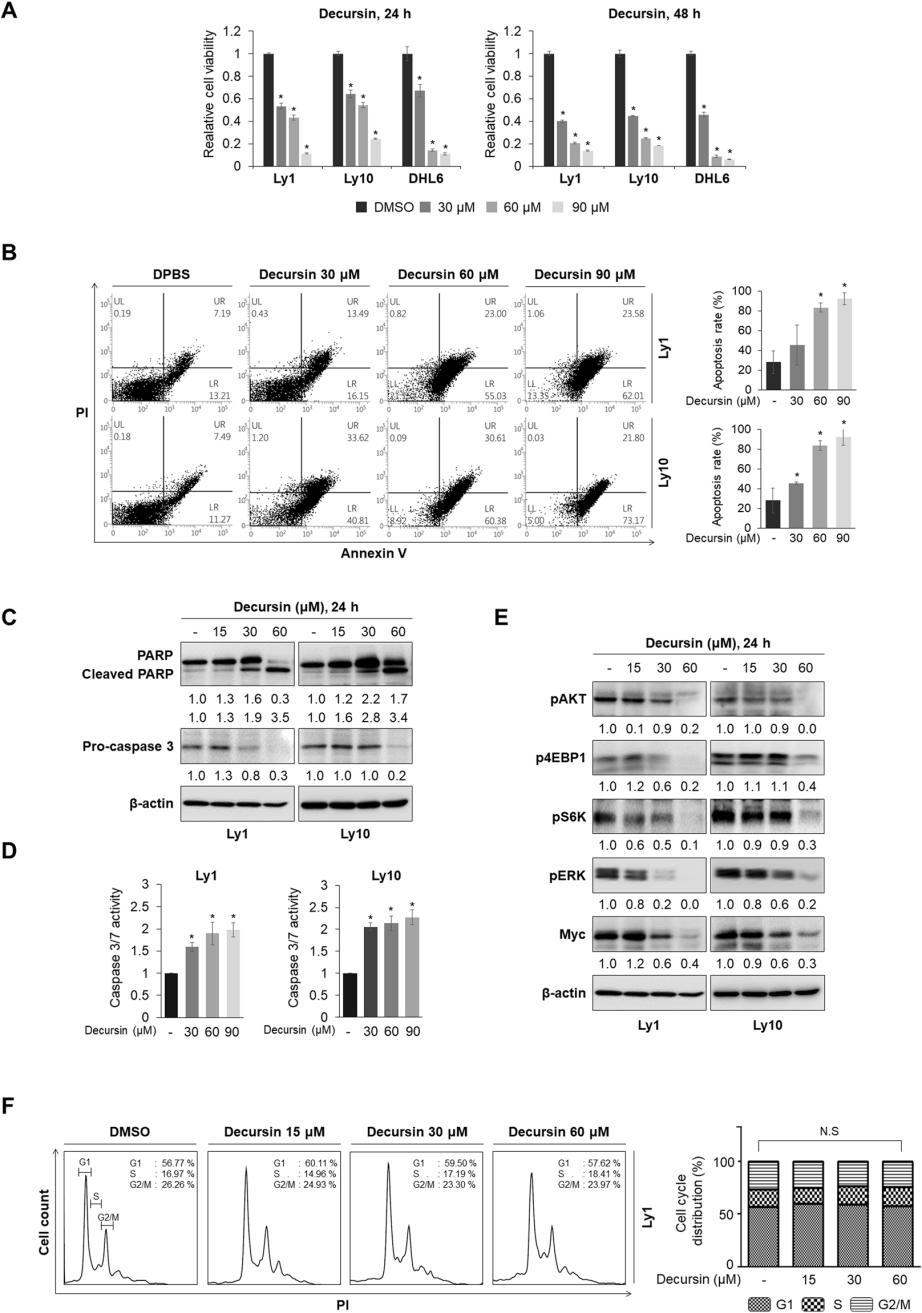
Decursin induces apoptosis in DLBCL cell lines. (**A**) Cell viability assays were conducted after Ly1 and Ly10 cells were treated with decursin (0, 30, 60, or 90 μM) for 24 and 48 h. Decursin inhibited cell survival in a dose-dependent manner. MTS assay was conducted in triplicate, and representative results are shown. Statistical significance was calculated using a two-tailed Student’s *t* test (*p < 0.05). (**B**) Decursin treatment (0, 30, 60, or 90 μM) for 24 h increased apoptotic populations in Ly1 and Ly10 cells when analyzed by flow cytometry after staining with Annexin V-FITC and PI. Representative data from at least three independent experiments are shown. A two-tailed Student’s *t* test is used to calculate statistical significance (*p < 0.05). (**C**) Cells were treated with decursin (0, 15, 30, or 60 μM) for 24 h. Cell lysates were subjected to western blotting with antibodies against PARP, cleaved PARP, and pro-caspase 3. β-actin was used as a loading control; representative results are shown. (**D**) Caspase 3/7 activities were detected by a bioluminescent assay in Ly1 and Ly10 cells after treatment with decursin (0, 30, 60 or 90 μM) for 24 h. Representative data from at least three independent experiments are shown. (**E**) Ly1 and Ly10 cells were treated with decursin (0, 15, 30, or 60 μM) for 24 h. Myc protein levels and levels of pAKT, pS6K, p4EBP1, and pERK were detected by western blotting. β-actin was used as a loading control. (**F**) Cell cycle analysis after staining with PI was performed in DLBCL cells treated with decursin (0, 15, 30, or 60 μM) for 24 h. Decursin had no effect on cell cycle progression.

It has been shown that decursin is the major chemical component in the extract of the AGN. To examine whether decursin mimics the cytotoxic effects of AGN in B lymphoma cells, three cell lines, Ly1, Ly10, and DHL6, were treated for 24 and 48 h. Our results suggest that cell viability was suppressed by decursin in a dose-dependent manner (Fig. 4A). Similar to AGN, flow cytometry analysis with Annexin V and PI showed that decursin increases the number of cells stained with annexin V and PI, indicating increased apoptosis in the cells (Fig. 4B). Consistent with these data, cleaved PARP level and caspase 3/7 activity were increased upon decursin treatment (Fig. 4C,D and Fig. S7). Additionally, western blot analysis indicated that decursin potently inhibits the phosphorylation of AKT, S6K, 4EBP1, and ERK; it also significantly downregulated Myc protein levels (Fig. 4E and Fig. S8). As is the case with AGN, decursin did not affect cell cycle progression (Fig. 4F).

### Myc is a downstream target of PI3K/AKT/mTOR and MAPK signaling pathways

**Figure 5:**
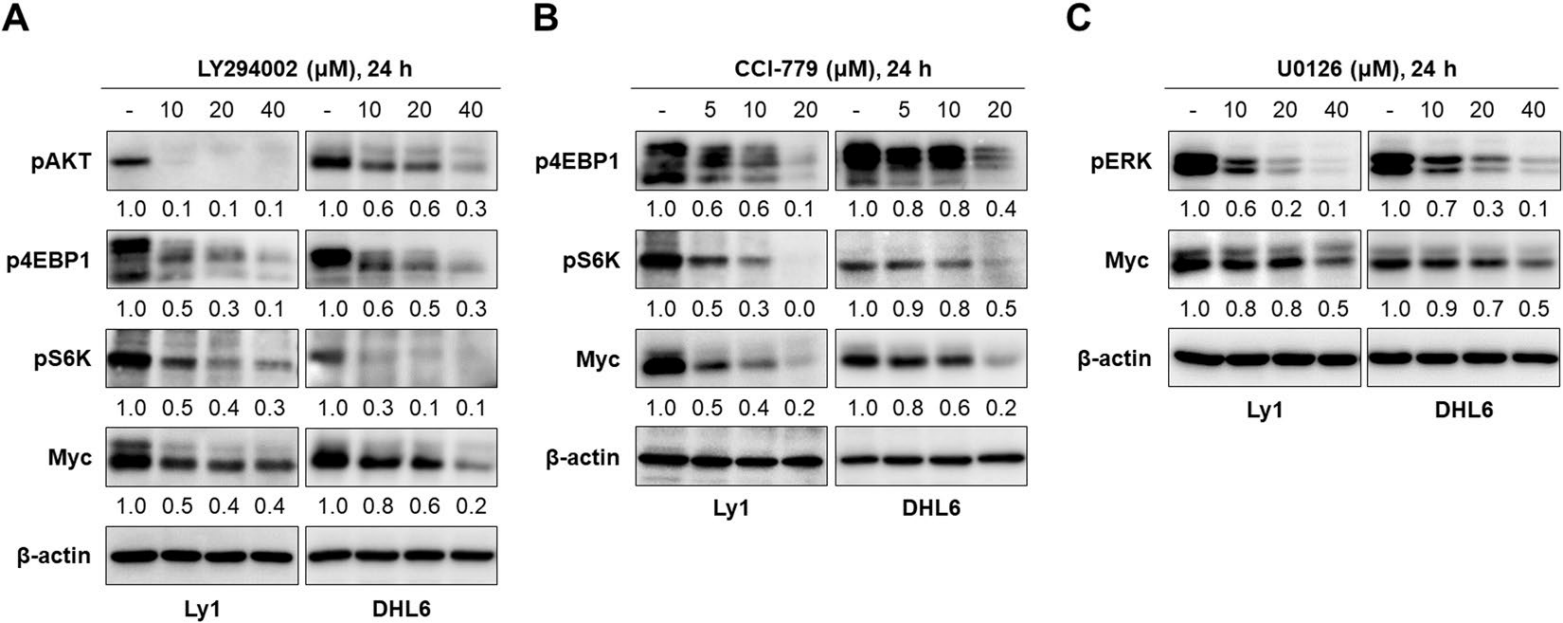
Myc is a downstream molecule of PI3K/AKT/mTOR and MAPK pathways. (**A**) The PI3K inhibitor LY294002 (0, 10, 20, 40 μM) was added in Ly1 and DHL6 DLBCL cells and the levels of pAKT, pS6K, p4EBP1, and Myc were analyzed by the western blotting. Inhibition of PI3K/AKT activities led to the downregulation of Myc. (**B**) Administration of CCI-779 (0, 5, 10, 20 μM), an inhibitor of mTOR signaling, downregulated the levels of pS6K, p4EBP1, and Myc. Blocking mTOR signaling downregulated Myc expression. (**C**) The MAPK inhibitor, U0126 (0, 10, 20, 40 μM), was added in the indicated cells, followed by the analysis of pERK and Myc levels by western blotting. Inhibition of MAPK activities diminished the expression of Myc.

Although the results in the previous sections suggest that a reduction in Myc levels by AGN (Fig. 2) and decursin (Fig. 4) occurs via inhibition of PI3K/AKT/mTOR and MAPK pathways, we cannot rule out the possibility that the down-regulation of Myc levels and these signaling pathways may simply be a correlation. If AGN and decursin regulated Myc levels via PI3K/AKT/mTOR and MAPK signaling pathways, inhibitors of these signals would recapitulate the effect of AGN and decursin. To directly test our hypothesis, we treated Ly1 and DHL6 DLBCL cells with LY294002, CCI-779, and U0126 to specifically inhibit PI3K/AKT, mTOR, and ERK signals, respectively. As shown in Fig. 5, administration of LY294002, CCI-779, and U0126 reproduced the effect of AGN and decursin on the expression of Myc; they suppressed the respective signaling pathway, which was evidenced by the downregulation of pAKT, p4EBP, and pERK, and inhibition of these signals led to a concomitant decrease in Myc levels (Fig. 5A–C and Fig. S9). These data suggest that Myc is a downstream molecule of these signaling pathways and AGN and decursin inhibit Myc expression via downregulation of PI3K/AKT/mTOR and ERK pathways.

### Co-treatment with decursin and a Myc inhibitor synergistically suppresses the viability of DLBCL cells

**Figure 6:**
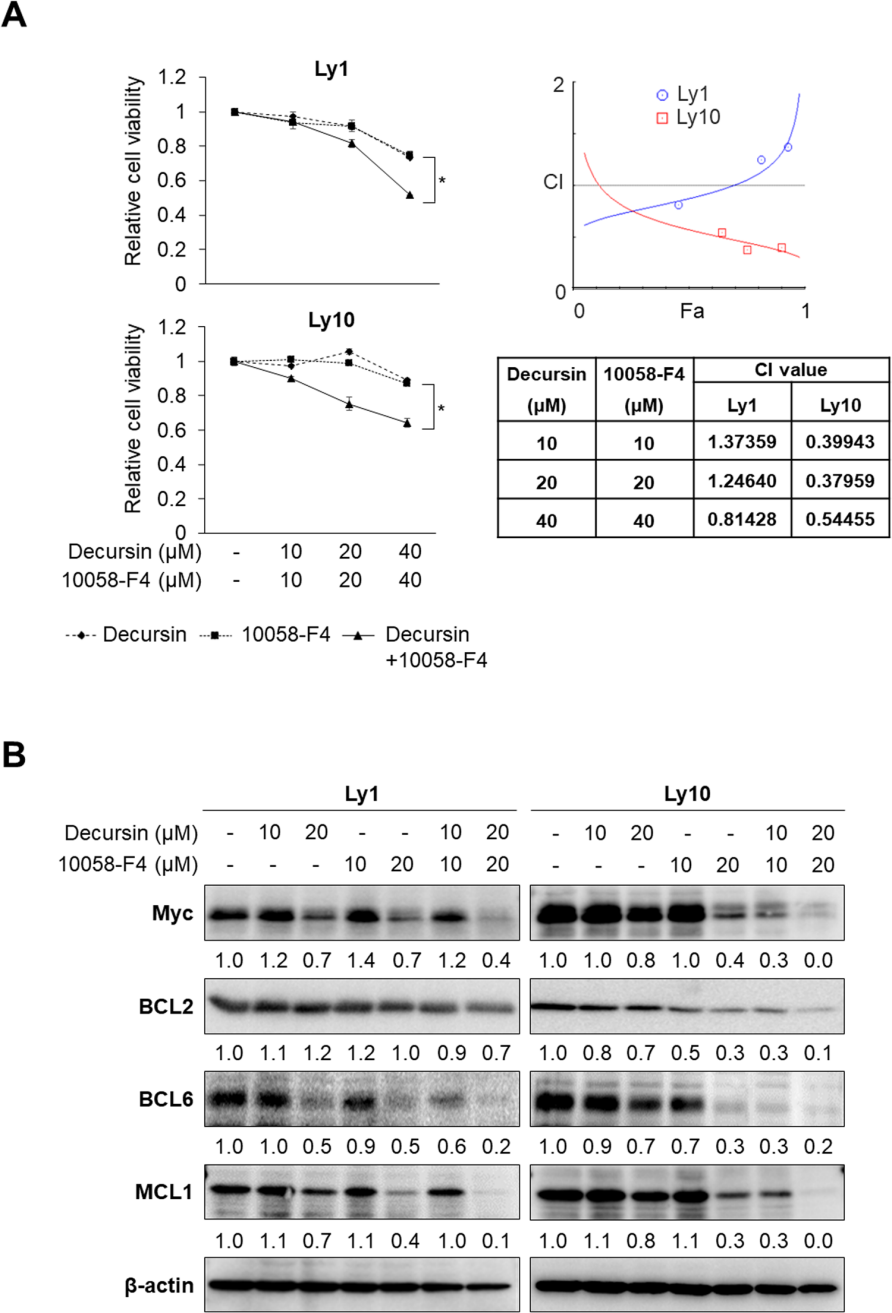
Co-treatment with decursin and 10058-F4 significantly suppresses cell survival. (**A**) Ly1 and Ly10 cells were exposed to decursin (0, 10, 20, or 40 μM) and/or 10058-F4 (0, 10, 20, or 40 μM) for 48 h, and MTS assays were performed to measure cell viability (Left panel). The statistical significance was calculated using a two-tailed Student’s *t* test (*p < 0.05). CI values were calculated using the Compusyn software (Right panel). (**B**) Western blot assays were conducted to determine the expression of Myc and anti-apoptotic proteins in two DLBCL cell lines following incubation with decursin and/or 10058-F4 for 48 h.

As mentioned earlier, combination treatments with AGN and Myc inhibitors inhibited cell growth, attenuated survival pathways, and decreased Myc protein levels better than single treatments. We examined whether decursin also exerts synergistic effects when combined with Myc inhibitors. To this end, Ly1 and Ly10 cells were exposed to decursin and 10058-F4, and we found that cell viability was reduced to a greater extent by the combination treatment than single treatments (Fig. 6A). CI values, calculated by the CompuSyn software, show synergism. Similar to AGN, we found that exposure to decursin in combination with 10058-F4 for 48 h significantly reduces the expression of BCL6, MCL1, BCL2, and Myc (Fig. 6B and Fig. S10). These data show that combination therapy with decursin and Myc inhibitors could be an effective therapeutic option.

### Anticancer effect of AGN/decursin is reproduced in Eμ-myc transgenic mice

**Figure 7:**
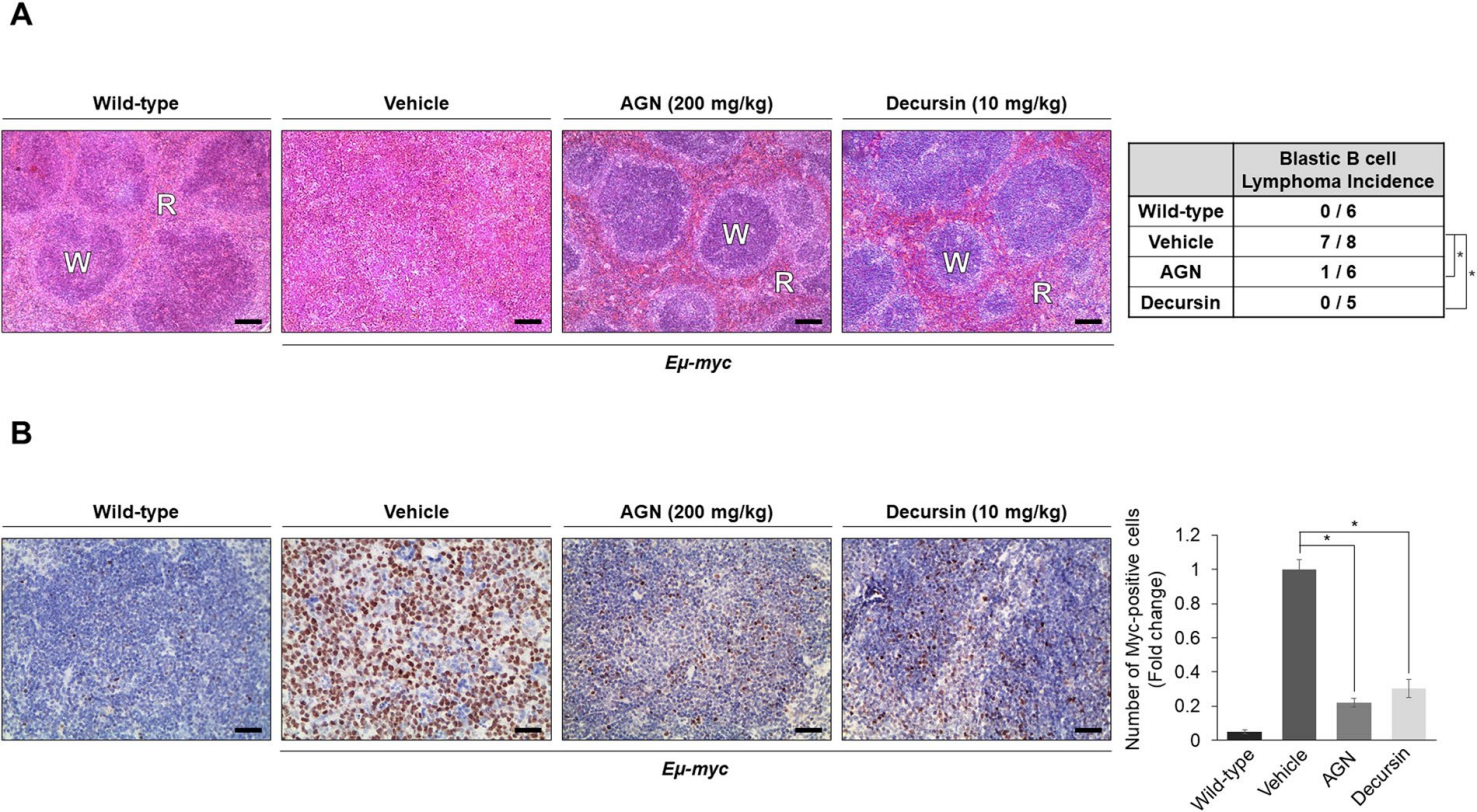
AGN and decursin attenuate lymphogenesis *in vivo*. (**A**) Eight-week old Eμ-myc transgenic mice were treated with vehicle or AGN (200 mg/kg) for eight weeks or decursin (10 mg/kg) for four weeks. Histological analyses of the spleen were performed using H&E staining. The microscopic architecture of spleen in vehicle-treated Eμ-myc mice was collapsed; however, it was restored to that of wild-type mice by the administration of AGN or decursin (*p < 0.05), Fisher’s exact test. Representative images of the H&E-stained spleen sections are shown. W and R represent white pulp and red pulp, respectively. Scale bar, 100 μm. (**B**) The spleens of wild-type or Eμ-myc mice were isolated after the treatments and analyzed by IHC with the antibody against Myc. Myc-positive cells were counted and cell counts are normalized to those in the vehicle group. The statistical significance was calculated using a two-tailed Student’s *t* test (*p < 0.05). Scale bar, 100 μm.

AGN/decursin exerted anti-tumor effects by inducing apoptosis *in vitro*. To assess whether these drugs also have anti-tumor potential *in vivo*, we used Eμ-myc transgenic mice, which is one of the most commonly used murine models for studying Myc-induced B-cell lymphoma. Eμ-myc transgenic mice have *Myc* gene that has been introduced in the lymphoid-specific IgH locus, and these mice exhibit significantly increased levels of pro-B and pre-B cells in splenocytes and bone marrow cells, resulting in B-cell lymphoma^11,14–16^. To determine the effect of AGN/decursin, we administered AGN (200 mg/kg) for eight weeks or decursin (10 mg/kg) for four weeks to Eμ-myc transgenic mice. We conducted H&E staining to examine spleen histopathology. In most of the vehicle-treated Eμ-myc mice, a collapse of the microscopic architecture of the spleen was observed; the boundaries between white pulp and red pulp disappeared, an indication of high grade blastic B-cell lymphomas^15,32^. However, treatment with AGN or decursin completely restored the normal appearance of the spleen (*p < 0.05, Fisher’s exact test), which is tightly related with a substantial inhibition of Myc expression (Fig. 7A,B). These results imply that AGN and decursin may be effective in the treatment of Myc-driven B-cell lymphoma.

## Discussion

Oriental herbal medicine is gaining popularity. AGN, one of the most commonly used herbal medicines, has been widely used to treat female diseases. Additionally, the anti-tumor activities of AGN and its major compounds decursin and decursinol angelate have been studied in various cancer cells, including human erythroleukemia, sarcoma, blood cancer, prostate cancer, and melanoma cells^7^. The anti-tumor activities of AGN and decursin were shown to be associated with inhibition of multiple survival signaling pathways, such as PI3K/AKT, ERK^33^, NF-κB^34^, and Wnt/β-catenin^35^. However, the efficacy of AGN/decursin in B-cell lymphoma and the underlying mechanisms are not established.

We demonstrated that AGN inhibits cell viability in three DLBCL cell lines (Ly1, Ly10, and DHL6) through the activation of caspase 3/7 by downregulating PI3K/AKT/mTOR and MAPK pathways (Figs 1 and 2). *Myc* proto-oncogene was found to be a critical downstream target of these pathways, mediating the cytotoxic activities of AGN/decursin. It has been recently reported that BCR-PI3K signaling plays a pivotal role in dysregulation of Myc expression and loss of BCR signaling renders cells sensitive to nutrient restriction in B lymphoma cells^36,37^, suggesting that BCR-Myc signaling is mainly responsible for the survival of this type of tumor, which we demonstrate can be efficiently suppressed by AGN/decursin. In addition, cytotoxic effect of AGN appears to be cancer cell-specific; it showed minimal cytotoxicity in normal splenocytes and bone marrow cells, which should be clinically beneficial.

Intriguingly, Myc inhibitors enhanced AGN/decursin-induced cytotoxicity in DLBCL cells, yielding synergistic anti-tumor effects. We suggest two explanations for the synergism between AGN/decursin and Myc inhibitors. First, low doses of single agents may not be enough to tip the balance towards cell death because of the presence of residual Myc expression that can be eliminated completely only by the administration of another agent^38^. Second, other survival factors, such as BCL2, MCL1, and BCL6, are significantly decreased by combination therapies compared with monotherapies. BCL2 is expressed in 40–80% of DLBCL cases. MCL1 is also frequently dysregulated in cancer, and contributes to resistance to BCL2 inhibitors^31,39,40^. This indicates that combination therapy can render cells that are resistant to BCL2 inhibitors susceptible to these drugs.

It is noteworthy that AGN elicits strong synergism, when combined with 10058-F4, while decursin shows only moderate synergism. These differential responses are correlated with our data demonstrating that AGN/10058-F4 has a greater inhibitory effect on Myc and the above-mentioned survival factors than decursin/10058-F4 (Figs 3 and 6). Another point to consider regarding this result is that AGN contains several compounds with anti-cancer effects, including N-butylidenephthalide and Z-ligustilide, in addition to decursin, although the first two are minor components^41^. This leaves open the possibility that the better synergistic effect from AGN may stem, at least in part, from these minor chemical constituents.

Drug resistance is almost inevitable when cancer patients are exposed to long-term chemotherapy, and combinatorial treatments are considered a way to re-sensitize the resistant cells to the therapy in a synergistic manner^42^. The efficacy of several BET inhibitors is being tested in clinical trials in patients with hematological malignancies^12^, although it is unfortunate that the development of resistance to these drugs are expected. In this regard, two recent papers identifying the mechanism underlying therapeutic resistance in acute myeloid leukemia (AML) as elevated Myc expression due to high Wnt signals are intriguing^43–45^, given the present study demonstrating that AGN and decursin efficiently downregulate Myc levels. This result suggests that they may be used to overcome resistance to JQ1 in AML and further studies are necessary in order to validate this idea.

Overall, our data indicate that AGN/decursin can efficiently block Myc expression by downregulating PI3K/AKT/mTOR and MAPK signaling pathways, critical downstream mediators of BCR, and exert synergistic effects against B-cell lymphoma when combined with Myc inhibitors. AGN and decursin hold significant potential for the treatment of this type of tumor; however, further investigations are warranted.

## Methods

### Ethics statement

The animal protocol used in this study was reviewed and approved by the Institutional Animal Care and Use Committee of Pusan National University. All experiments were performed in accordance with the guidelines and regulations set and approved by Pusan National University.

### Cell culture, antibodies, and reagents

Three human DLBCL cell lines (Ly1, Ly10, and DHL6) were cultured in RPMI medium (Gibco) supplemented with 10% fetal bovine serum (FBS, Hyclone), 1% HEPES buffer, 1% L-glutamine, and 1% penicillin/streptomycin at 37 °C in a CO_2_ incubator.

Primary antibodies against the following proteins were used for western blotting: PARP and cleaved PARP [Santa Cruz Biotechnology (SCB); sc-7150], caspase 3 (SCB; sc-7148), Myc (Abcam; ab34072), pAKT [S473; Cell Signaling Technology (CST); 9271], p4EBP1 (CST; 9459), pS6K (T389; CST; 9206), pERK 1/2 (CST; 9101), BCL2 (SCB; sc-7382), BCL6 (SCB; sc-858), MCL1 (SCB; sc-819), and β-actin (SCB; sc-47778).

AGN extracts were prepared as previously described^46^. Decursin [(Sigma; SML0786, for *in vitro* assays) or (ChemFaces; CFN98509, for *in vivo* experiments)], BET bromodomain inhibitor JQ1 (Abcam; ab141498), a c-Myc inhibitor 10058-F4 (Calbiochem; 475956), LY294002 (Calbiochem; 440202), CCI-779 (Sigma; PZ0020) and U0126 (Sigma; U120) were purchased from commercial sources.

### Cell viability (MTS) assays

Cytotoxicities of AGN/decursin and Myc inhibitors, alone or in combination, were determined using the CellTiter 96 Aqueous Non-Radioactive Cell Proliferation Assay (MTS; Promega, Madison, WI, USA,). Cells were plated in 96-well microplates at a density of 5 × 10^4^ DLBCL cells or 5 × 10^5^ normal cells (splenocytes and bone marrow cells from wild-type mice) per well and treated with AGN (0, 2, 3, or 4 mg/ml), decursin (0, 30, 60, or 90 μM), JQ1 (0–800 nM), 10058-F4 (0–80 μM), or their combinations. After incubation for 24 and 48 h, the MTS reagent was added, and the cells were further incubated for 2 h. Optical density at 450 nm was measured using GloMax^TM^ Microplate multi-mode reader (Promega). MTS assays were conducted in triplicates and repeated independently three times.

### Immunoblotting

To investigate protein levels, relevant cells were seeded in 12-well plates at a density of 8 × 10^5^ per well and were treated with the previously mentioned drugs. After incubation, the cells were harvested and lysed in a RIPA lysis buffer (iNtRON Biotechnology) containing Na-vanadate (1 mM), β-glycerol phosphate (50 mM), a protease inhibitor cocktail (G-Biosciences), EDTA (5 mM), and β-mercaptoethanol (142 mM; BioWORLD). Samples were boiled at 100 °C for 10 min after adding 5× sample buffer and were loaded on polyacrylamide gels. Separated proteins were transferred to PVDF membranes using the Mini Trans-Blot® Cell and Critreion^TM^ Blotter (Bio-Rad), were blocked in 1% bovine serum albumin (MP Biomedicals), dissolved in Tris-buffered saline containing Tween 20 (TBST), and probed with primary antibodies overnight at 4 °C. After washing three times for 5 min each with TBST, the blots were exposed to an anti-mouse/rabbit secondary antibody (Bethyl) for 1 h, and the membranes were washed for 10 min in TBST three times. Protein bands were detected using a chemiluminescent substrate [EzWestLumi plus (Atto) or D-plus^TM^ ECL Pico System (DonginLS)] and visualized using the Luminograph II (Atto).

### Apoptosis assays

Cells were seeded in 12-well plates (8 × 10^5^/well), treated with AGN (0, 2, or 4 mg/ml) or decursin (0, 30, 60, or 90 μM) for 24 h, and stained with a FITC Annexin V apoptosis detection kit I (BD Biosciences) according to the manufacturer’s instructions, followed by analysis using a flow cytometer (FACSVerse, BD Biosciences). For the detection of caspase 3/7 activation, we followed the manufacturer’s instructions. Briefly, DLBCL cells were seeded in 96-well white opaque plates at a density of 1.5 × 10^4^/well and treated with AGN or decursin for 24 h. Caspase-Glo® 3/7 Assay reagent (Promega; G8090) was added to the wells followed by 1 h incubation at room temperature, and luminescence was measured using GloMax^TM^ Microplate multi-mode reader (Promega).

### Cell cycle analysis

DLBCL cells (1.5 × 10^6^/well) were seeded and treated with AGN (0, 1, 2, or 3 mg/ml) or decursin (0, 15, 30, or 60 μM) for 24 h. Cells were harvested, washed with 1 × PBS twice, and fixed in 75% ethanol overnight at 4 °C. After centrifugation (2,000 rpm, 5 min, 21 °C), the cells were stained with a PI solution (40 μg/ml of PI in PBS with 0.1% TritonX and 100 μg/ml RNase A) for 1 h at room temperature and analyzed using BD FACS Canto II software (BD Biosciences). Data acquisition and analysis were performed using the FlowJO software.

### Eμ-myc transgenic mice

Mice bearing the Eμ-myc transgene were obtained from the Jackson Laboratory (Bar Harbor, ME, USA). The strain was maintained by breeding hemizygous Eμ-myc transgenic males with wild-type C57BL/6 females. To test the efficacy of AGN and decursin, the mice were administered AGN (200 mg/kg) for eight weeks or decursin (10 mg/kg, ChemFaces) for four weeks. After sacrificing the mice, H&E staining were performed as previously described^47^. For the immunohistochemical (IHC) staining of Myc in the spleen tissues, a polyclonal anti-Myc primary antibody (Abcam; ab34072), suitably diluted with a protein diluent (Dako), and a polymer-horseradish peroxidase anti-rabbit (Dako) secondary antibody were used followed by 3,3- diaminobenzidine treatment to visualize the proteins. IHC and H&E-stained samples were examined at 100× magnification (scale bar, 100 μm) with an Olympus CX31 microscope (Olympus Corporation, Tokyo, Japan). The representative images were photographed using a digital photomicrographic camera attachment Moticam 2000 (Motic Co. Ltd., Kowloon, Fujian, China) and Motic Images Plus 2.0 software (Motic Co. Ltd., Kowloon, Fujian, China) and then assembled with PowerPoint software (Microsoft, Redmond, WA, U.S.A).

### Combination index (CI) values and statistical analysis

To determine the synergistic interaction between AGN/decursin and Myc inhibitors, MTS assays were performed in which the concentrations of drugs were gradually increased while maintaining a constant ratio of the drugs. CI values were calculated using the CompuSyn software. Synergy levels are as follows: <0.1, very strong synergism; 0.1–0.3, strong synergism; 0.3–0.9, synergism; 0.90–1.10, nearly additive; and >1.10, antagonism (modified from Chou, 2006^48^). Data are presented as the mean ± SD. Statistically significant differences were calculated by the Student’s *t* test using Microsoft Office Excel and Prism software (GraphPad). All experiments were repeated at least three times independently. For statistical analysis of high grade blastic B-cell lymphoma incidence in Eμ-myc transgenic mice in Fig. 7A, Fisher’s exact test was conducted.

## Electronic supplementary material

Supplementary figures
