# Supplementary material for: Angelica gigas Nakai and Decursin Downregulate Myc Expression to Promote Cell Death in B-cell Lymphoma
Source: Sci Rep. 2018 Jul 12;8:10590. doi: 10.1038/s41598-018-28619-z (PMC6043616; doi:10.1038/s41598-018-28619-z)
Supplement: Supplementary file 1 — Supplementary figures [file 41598_2018_28619_MOESM1_ESM.pdf]

SUPPLEMENTARY INFORMATION

Angelica gigas Nakai and Decursin Downregulate Myc Expression to Promote Cell Death in B-cell Lymphoma

Eungyoung Kim[1], Jehyun Nam[1], Woochul Chang[2], Ismayil S. Zulfugarov[3,4,5], Zhanna M. Okhlopkova[4], Daniil Olennikov[6], Nadezhda K. Chirikova[4], and Sang-Woo Kim[1,7*]

[1]Department of Integrated Biological Science, [2]Department of Biology Education, [3]Department of Molecular Biology, Pusan National University, Pusan 46241, Republic of Korea; [4]Department of Biology, North-Eastern Federal University, 58 Belinsky Str., Yakutsk 677027, Russia; [5]Institute of Molecular Biology and Biotechnology, Azerbaijan National Academy of Sciences, Matbuat Avenue 2a, Baku AZ 1073, Azerbaijan; [6]Institute of General and Experimental Biology, Sakh'yanovoy Str. 6, Ulan-Ude, Russia; [7]Department of Biological Sciences, Pusan National University, Pusan 46241, Republic of Korea

[*] Correspondence to:

Sang-Woo Kim, Ph.D.

Department of Biological Sciences, Pusan National University, Pusan 46241, Republic of Korea

E-mail: kimsw@pusan.ac.kr

Phone: 82-51-510-2260

Supplementary Figure S1. Full size blots of Figure1D

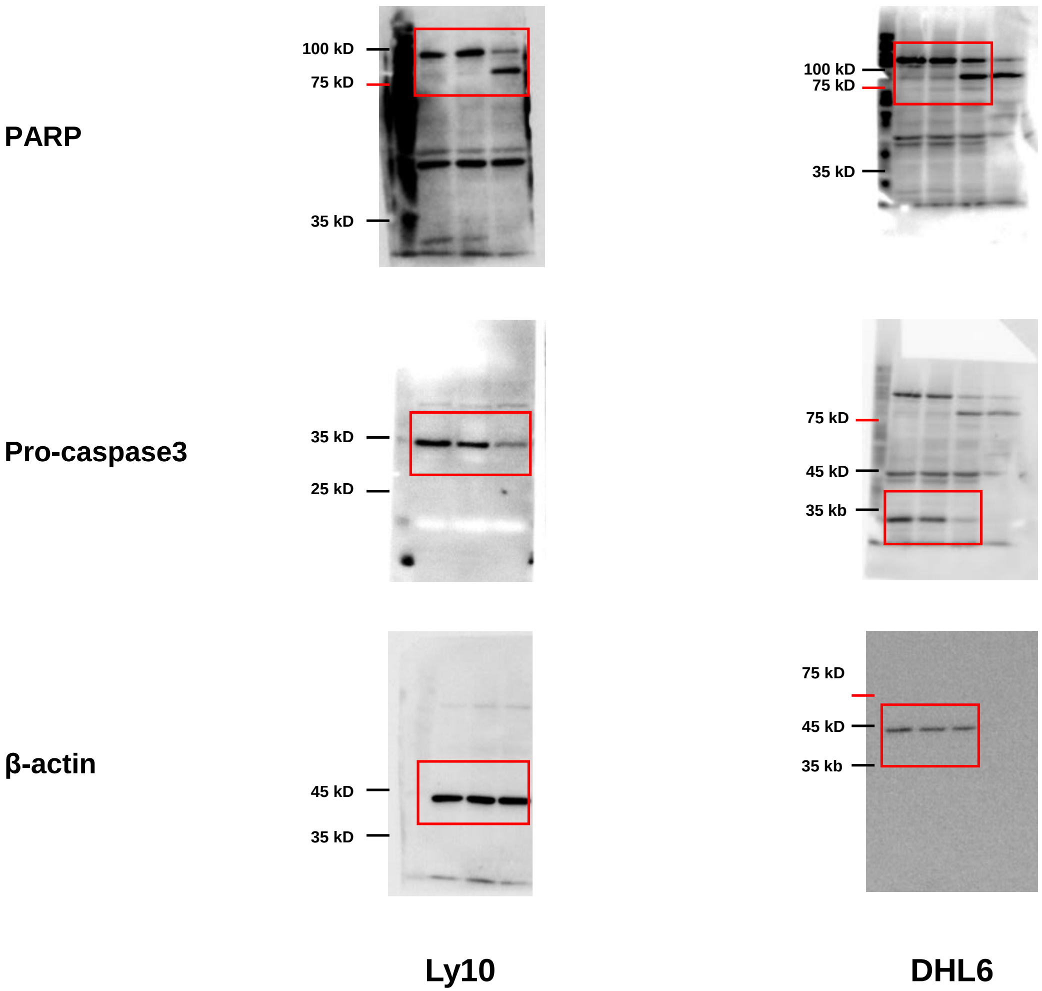

Supplementary Figure S2A. Full size blots of Figure2A – Ly1

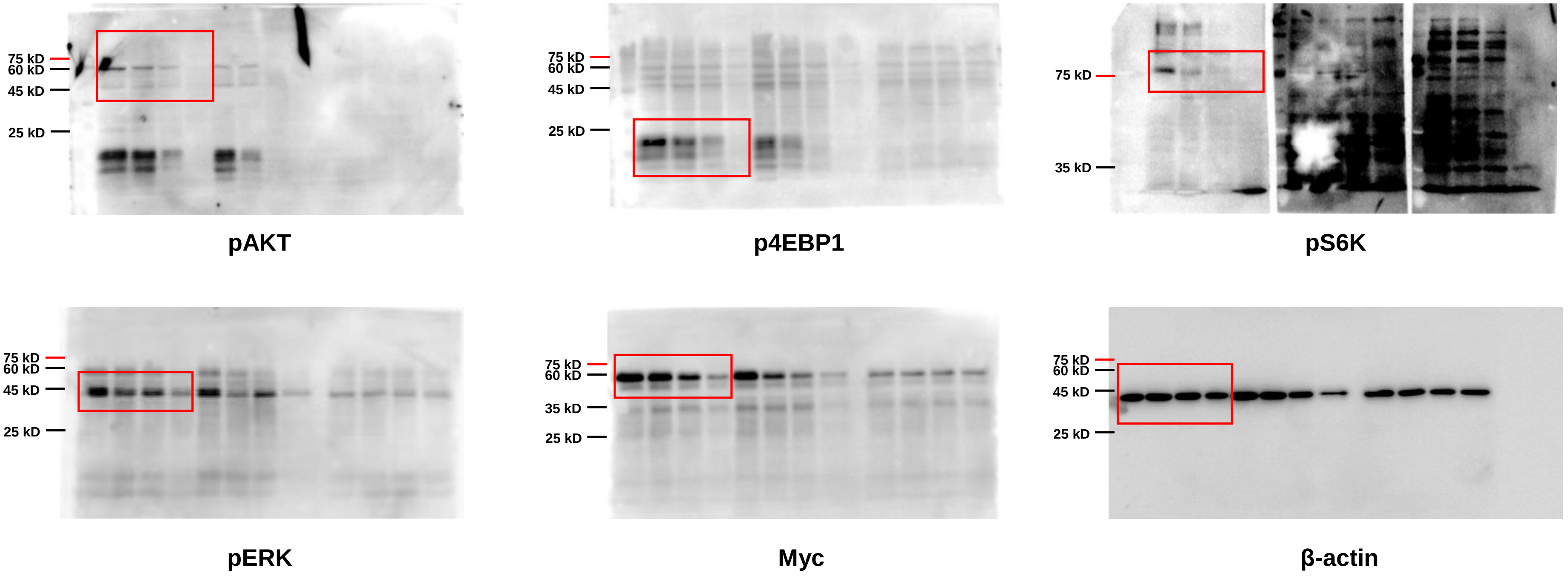

Ly1

Supplementary Figure S2B. Full size blots of Figure2A – Ly10

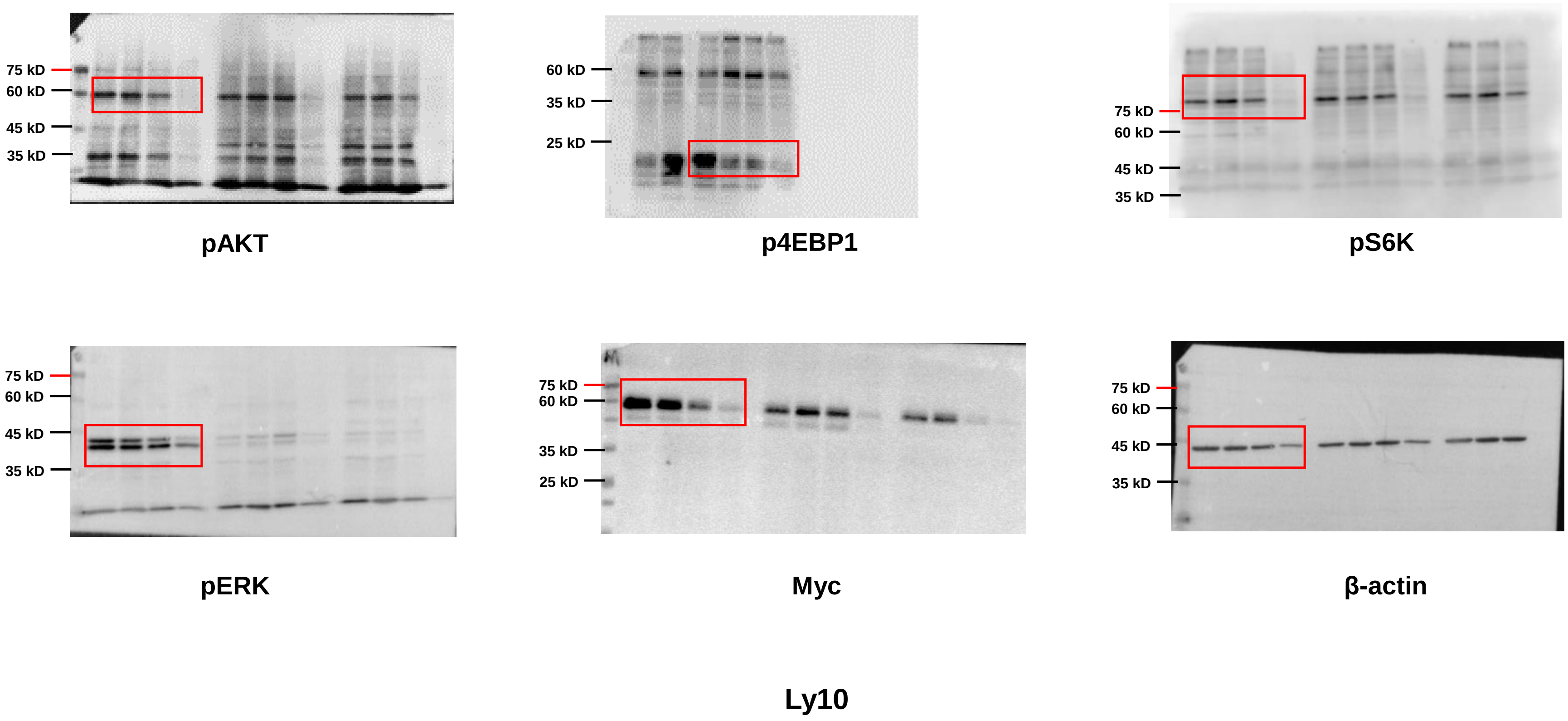

Supplementary Figure S2C. Full size blots of Figure2A – DHL6

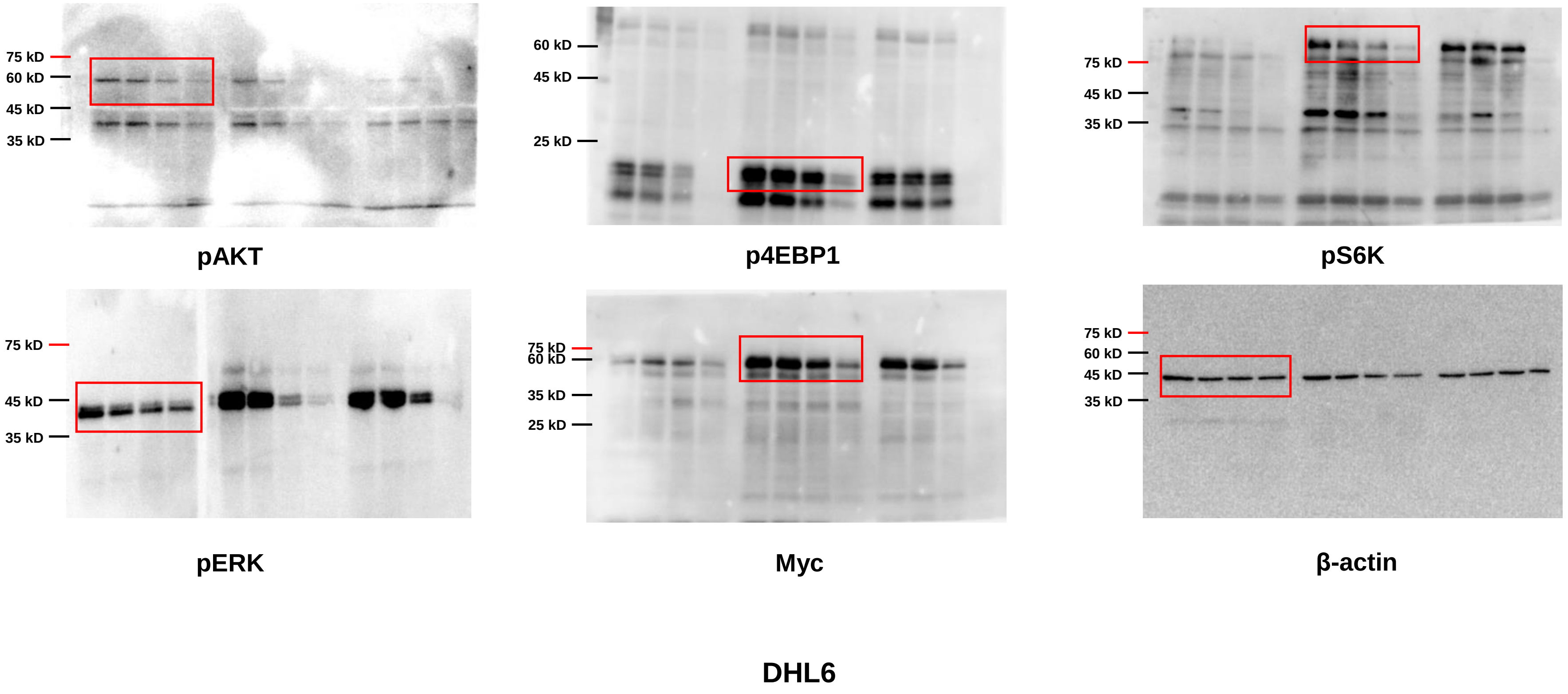

Supplementary Figure S3. Full size blots of Figure2B

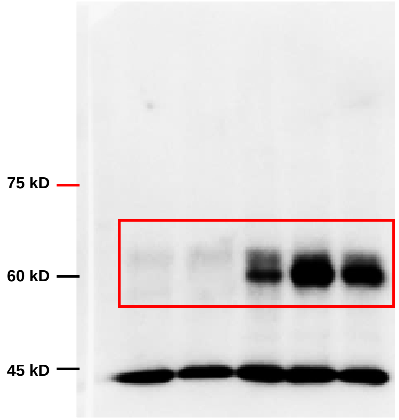

Myc

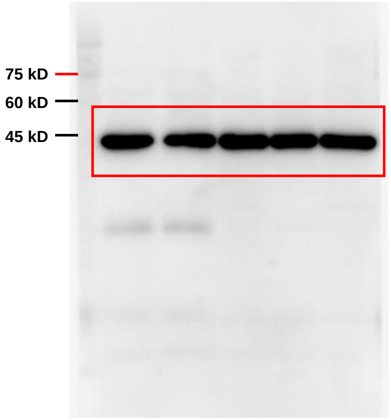

β-actin

Supplementary Figure S4A. Full size blots of Figure2C - Upper panel

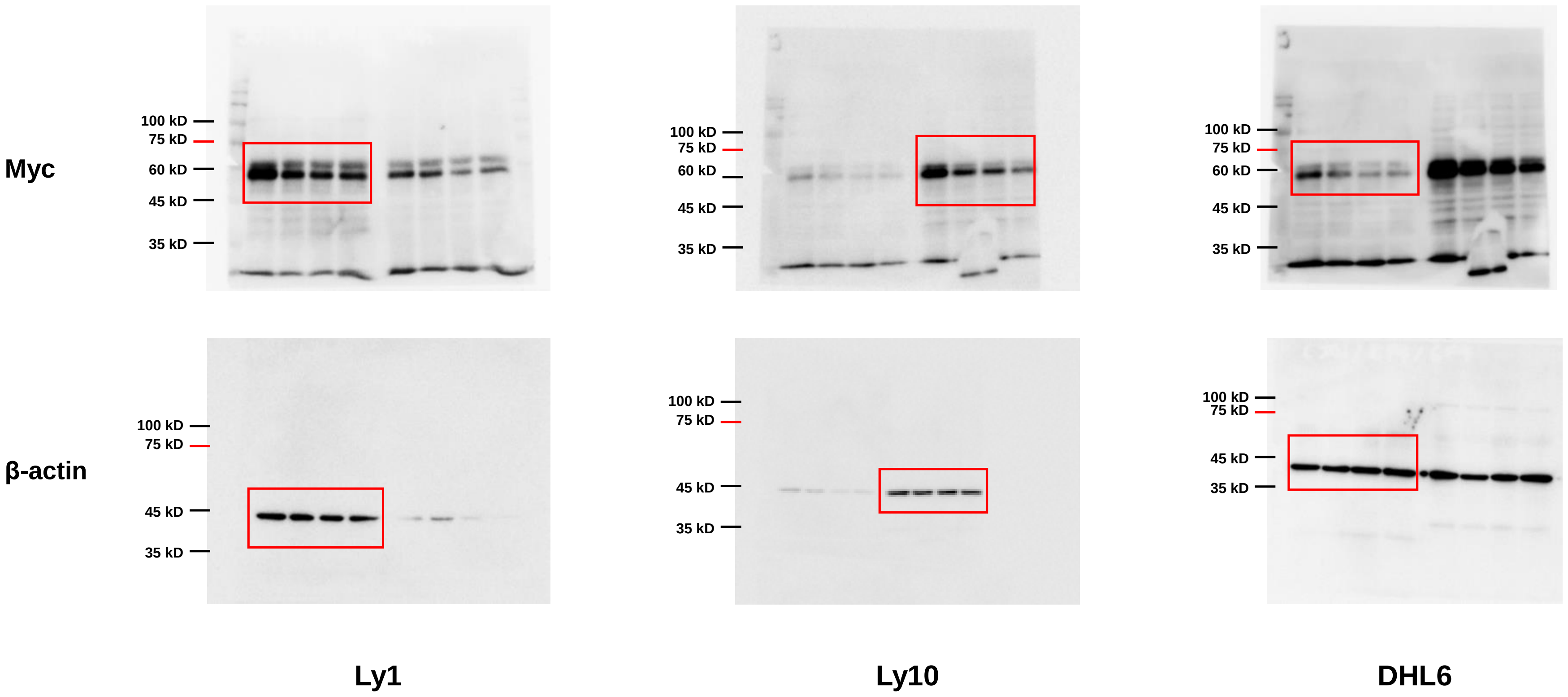

Supplementary Figure S4B. Full size blots of Figure2C - Bottom panel

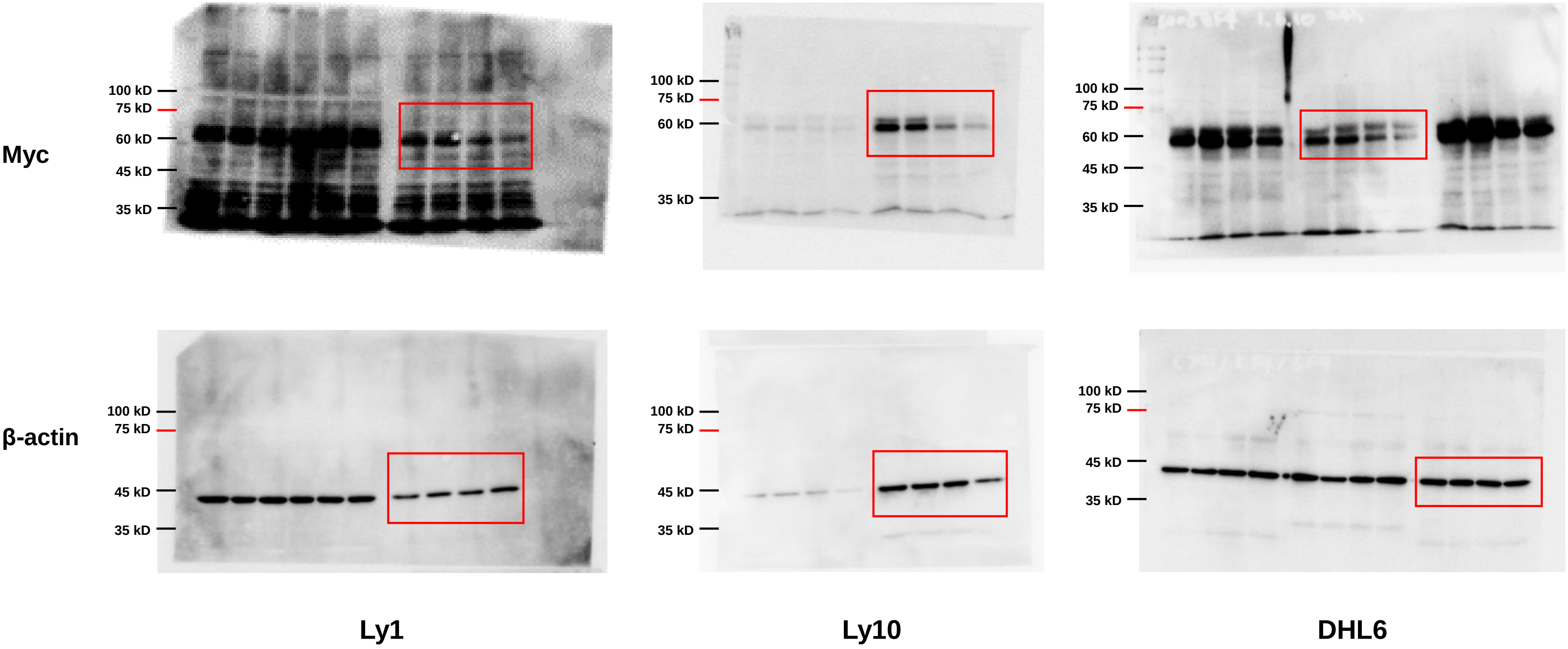

Supplementary Figure S5A. Full size blots of Figure3C - Ly1

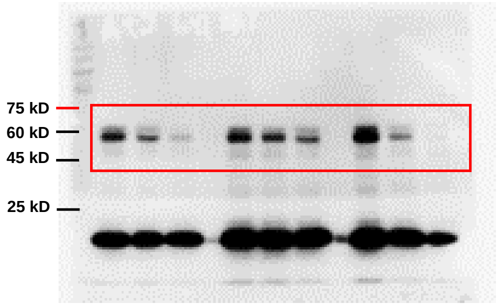

Myc

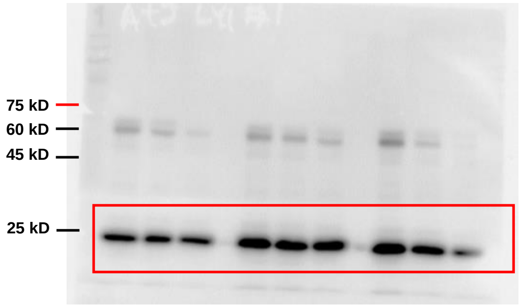

BCL2

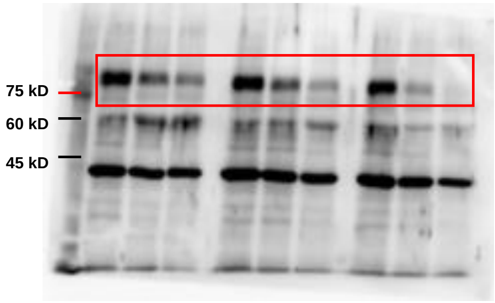

BCL6

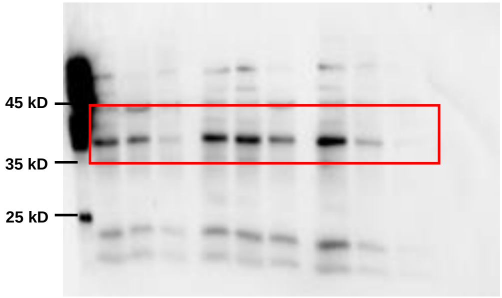

MCL1

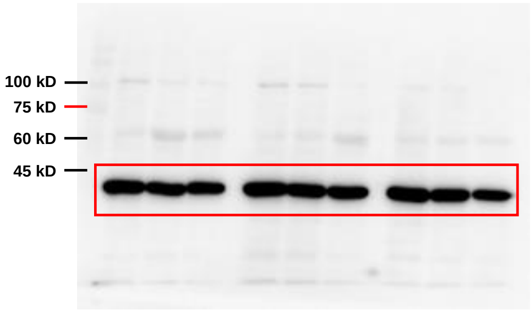

β-actin

Ly1

Supplementary Figure S5B. Full size blots of Figure3C - Ly10

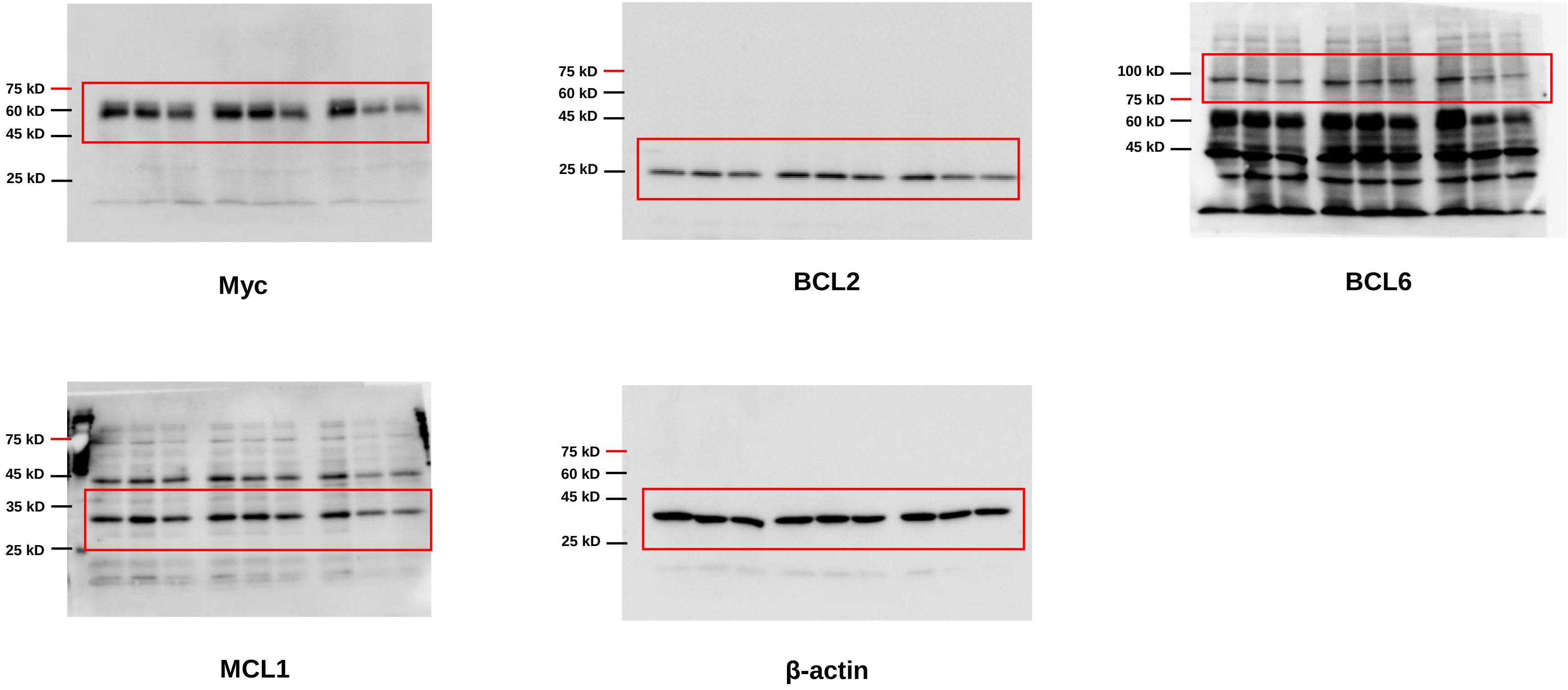

Ly10

Supplementary Figure S6A. Full size blots of Figure3D - Ly1

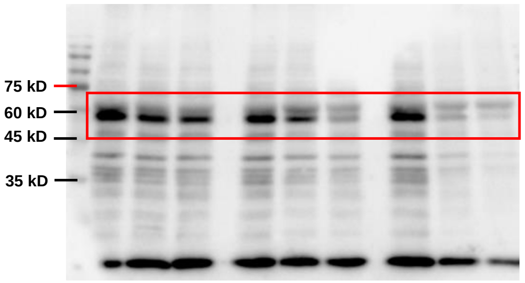

Myc

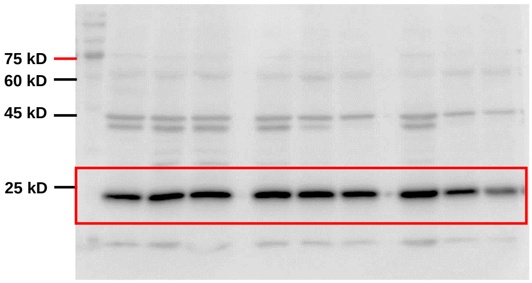

BCL2

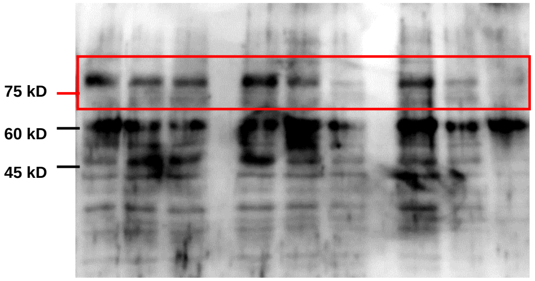

BCL6

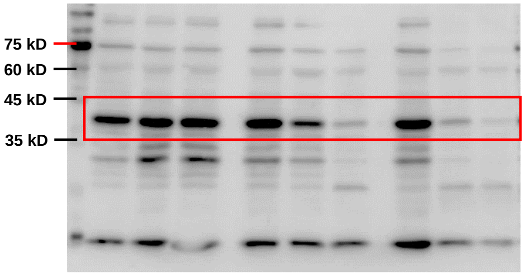

MCL1

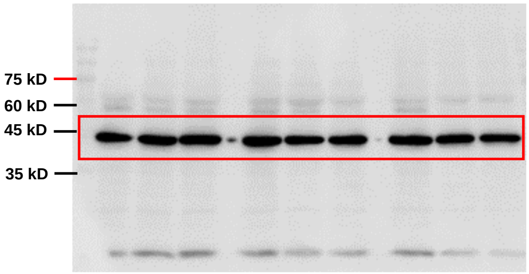

β-actin

Ly1

Supplementary Figure S6B. Full size blots of Figure3D - Ly10

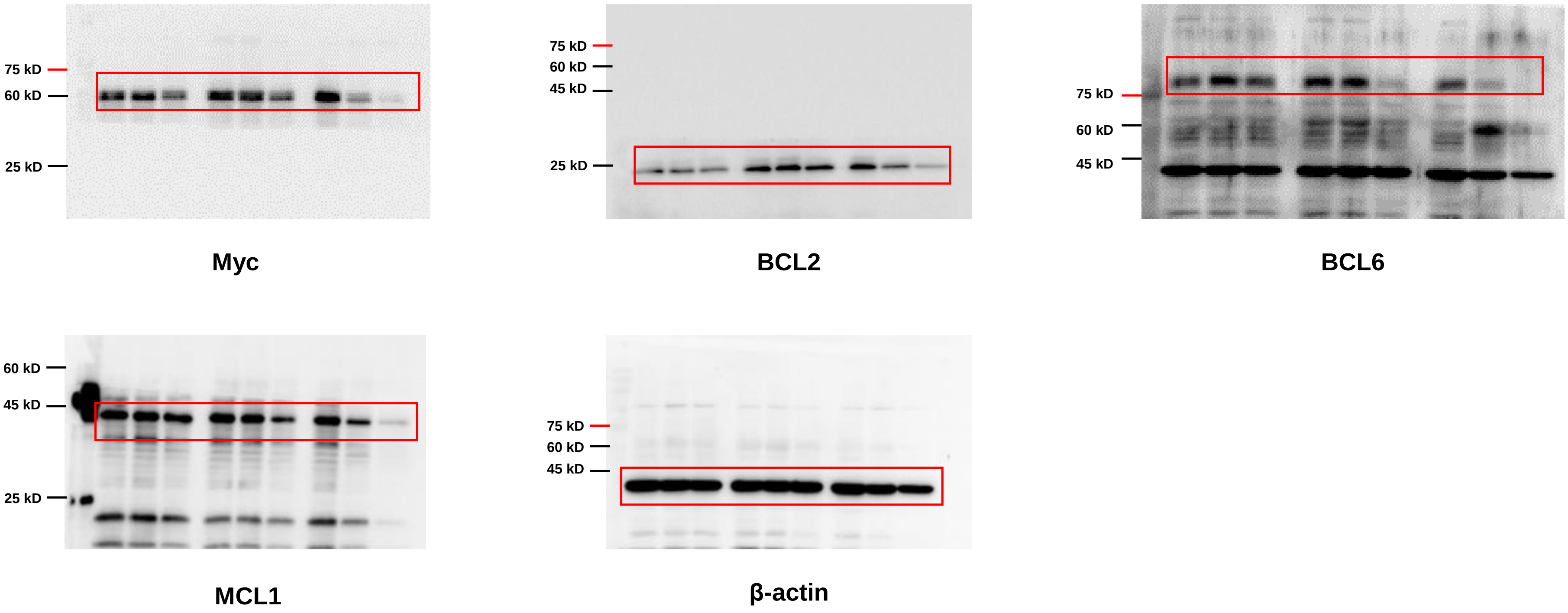

Ly10

Supplementary Figure S7. Full size blots of Figure4C

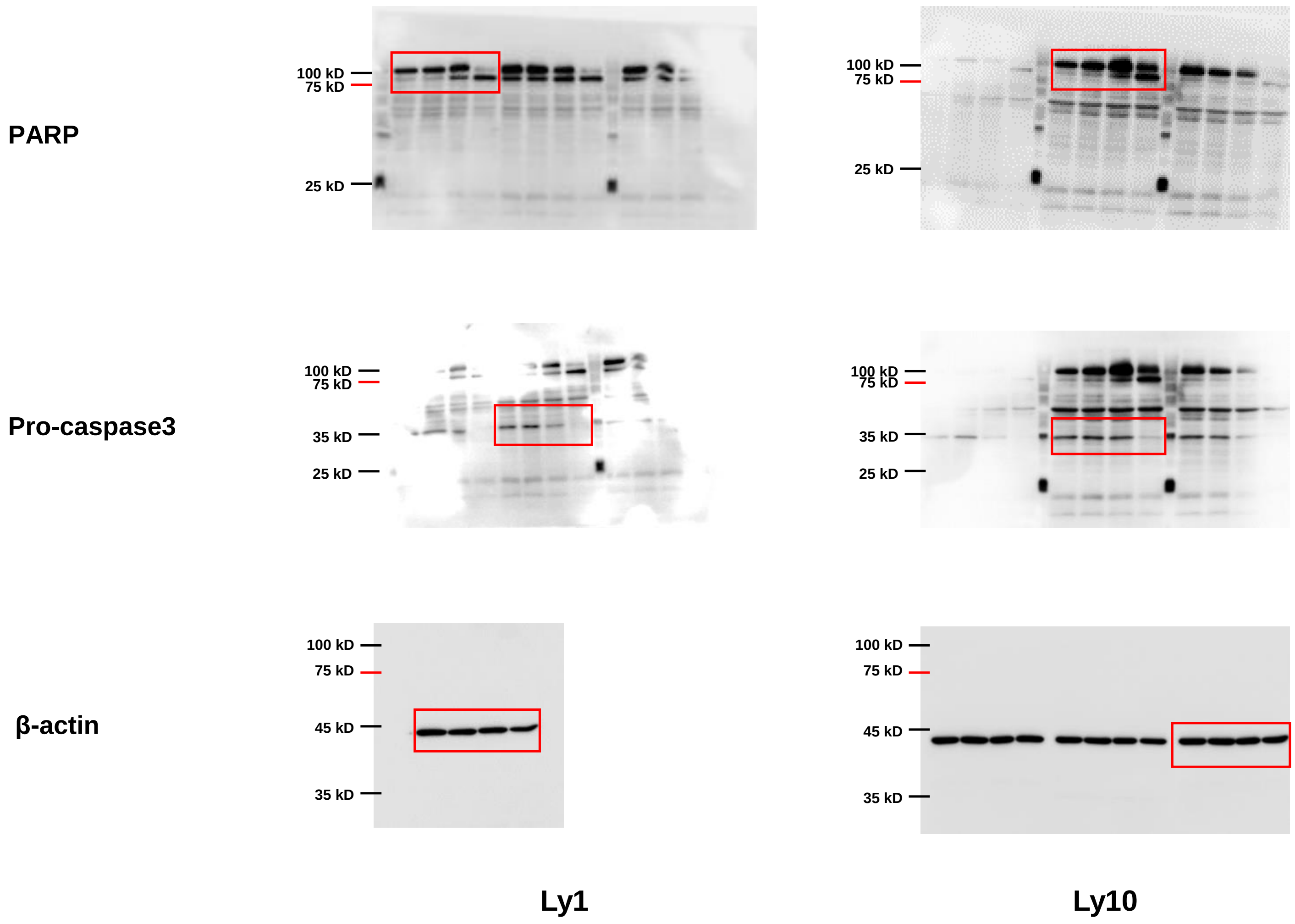

Supplementary Figure S8A. Full size blots of Figure4E - Ly1

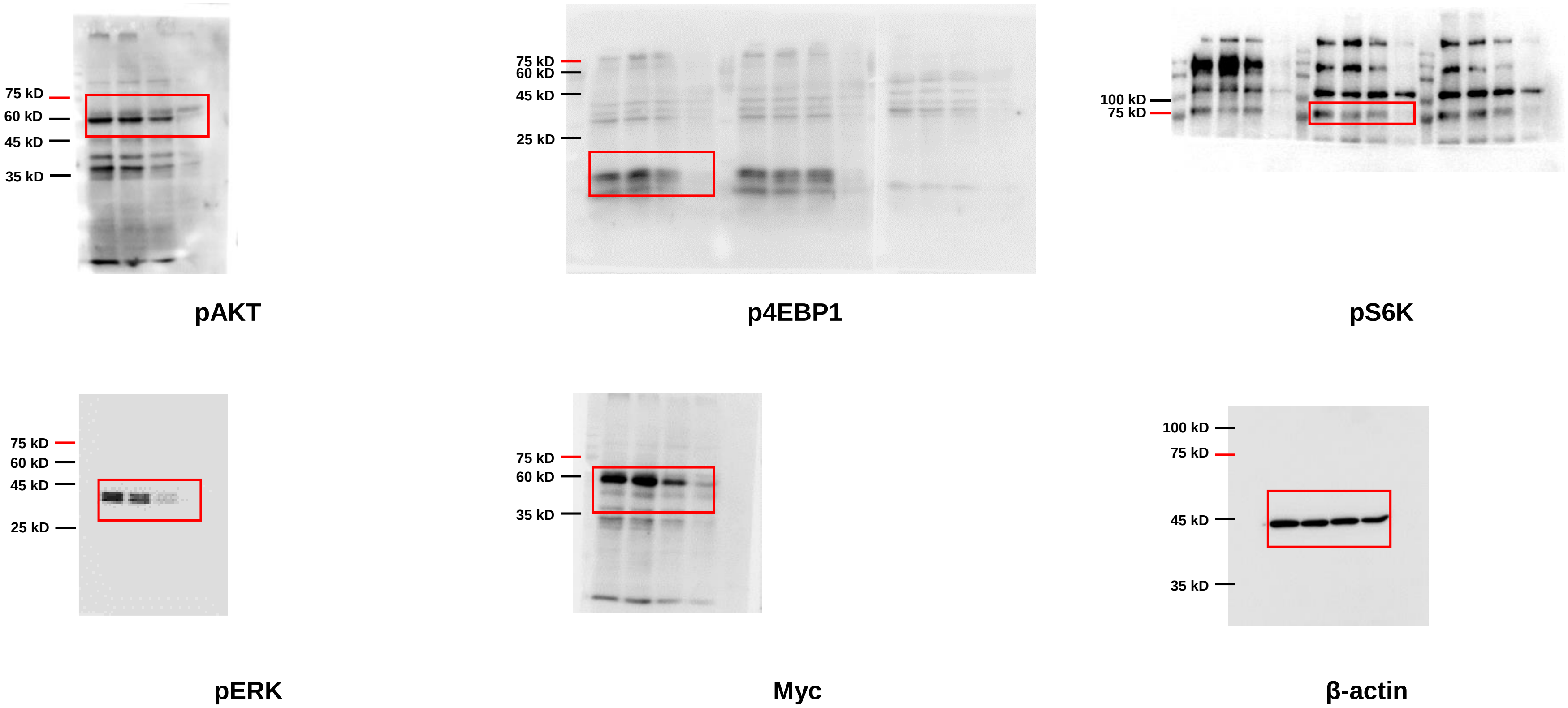

Ly1

Supplementary Figure S8B. Full size blots of Figure4E - Ly10

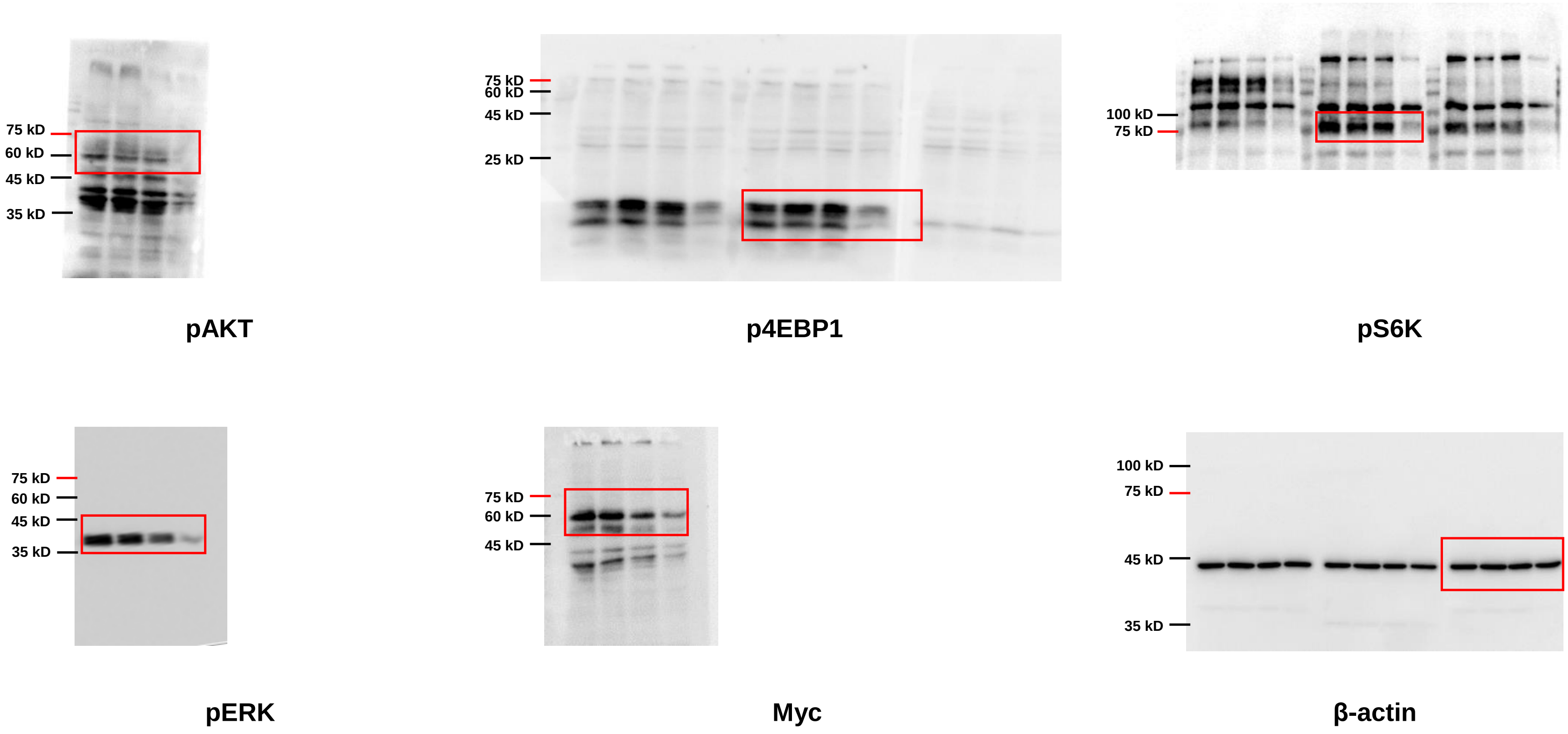

Ly10

Supplementary Figure S9A. Full size blots of Figure5A – Ly1

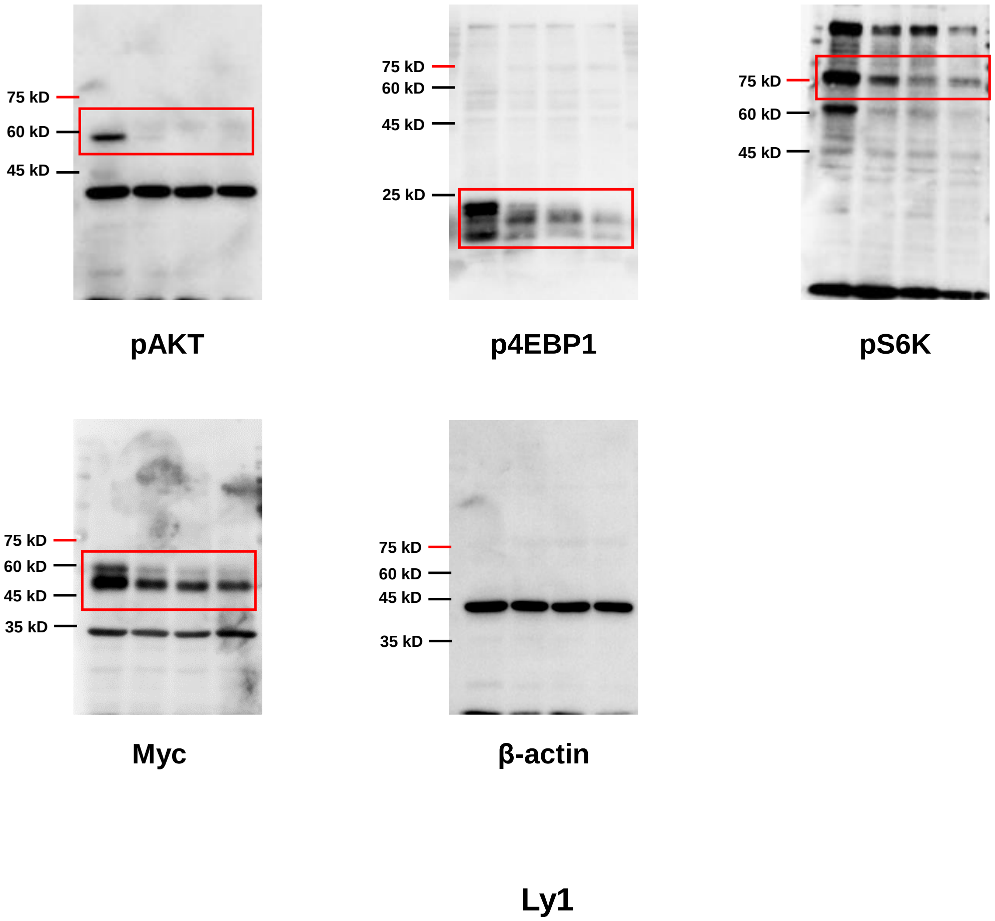

Supplementary Figure S9B. Full size blots of Figure5A – DHL6

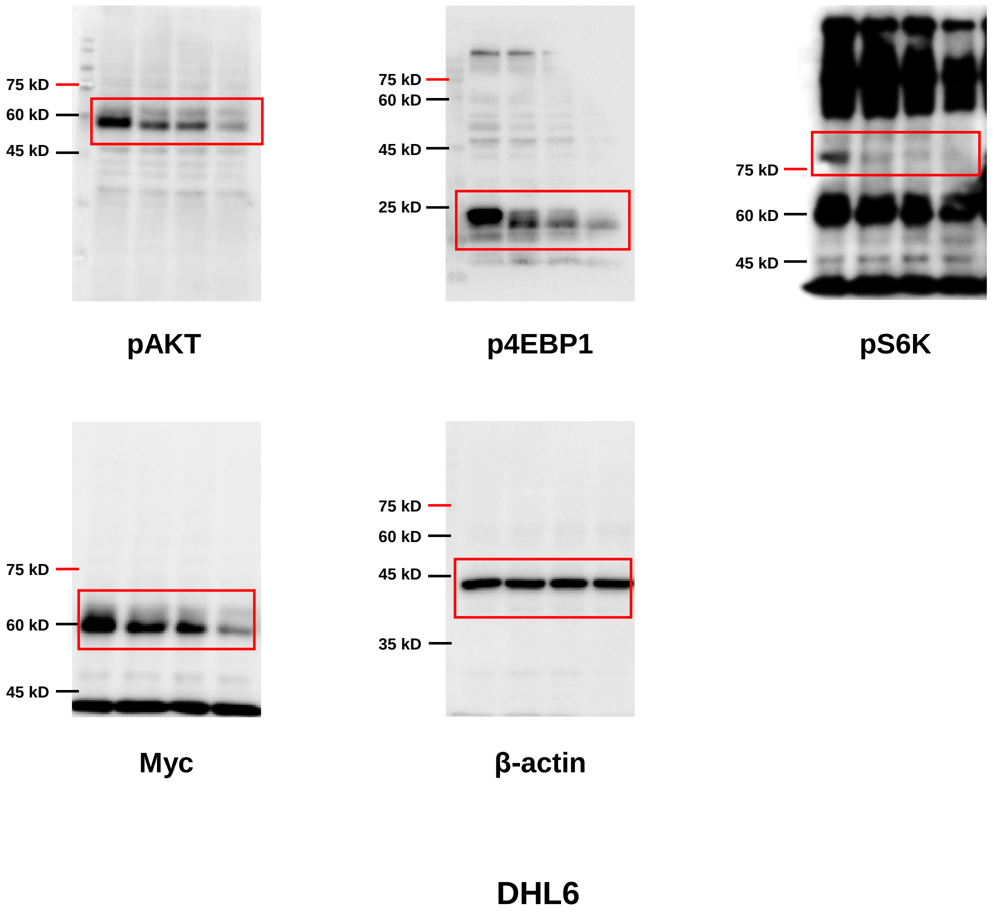

Supplementary Figure S9C. Full size blots of Figure5B – Ly1

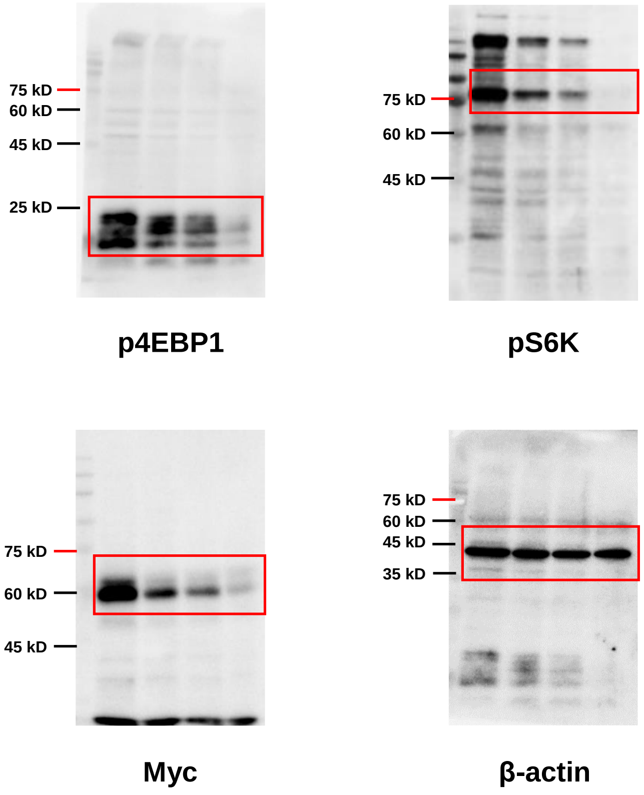

Ly1

Supplementary Figure S9D. Full size blots of Figure5B – DHL6

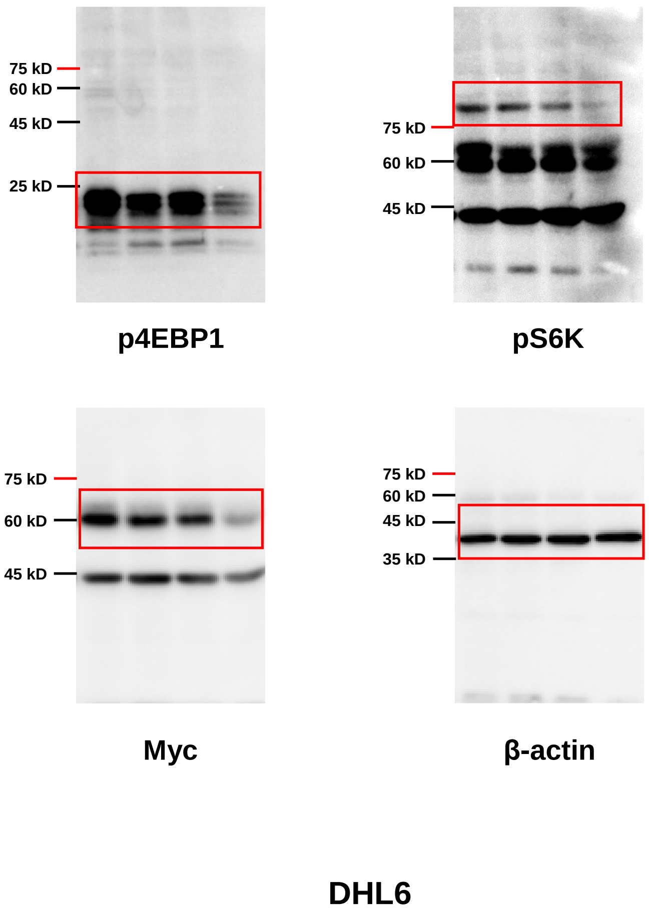

Supplementary Figure S9E. Full size blots of Figure5C – Ly1

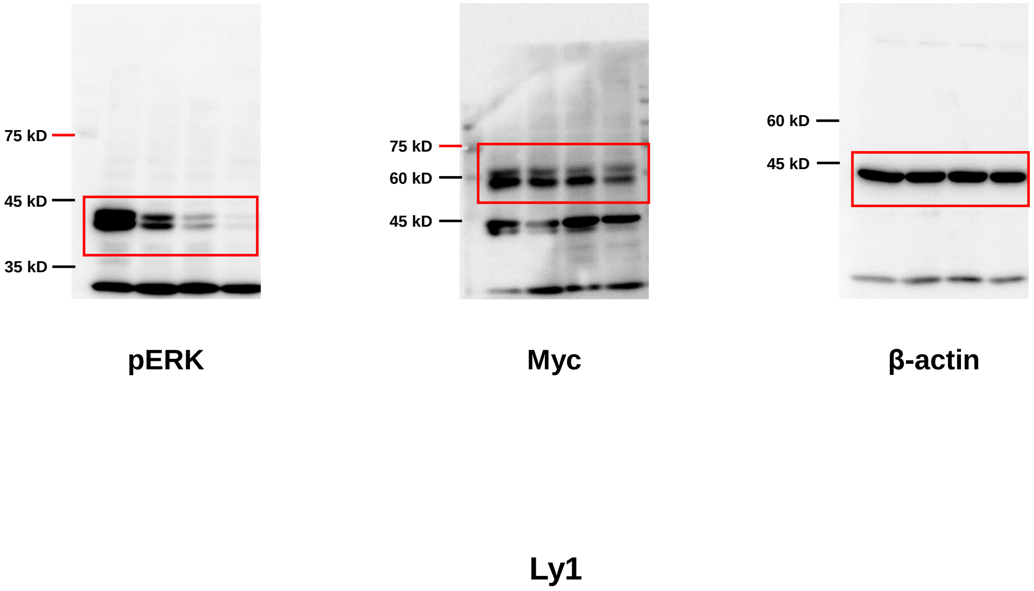

Supplementary Figure S9F. Full size blots of Figure5C – DHL6

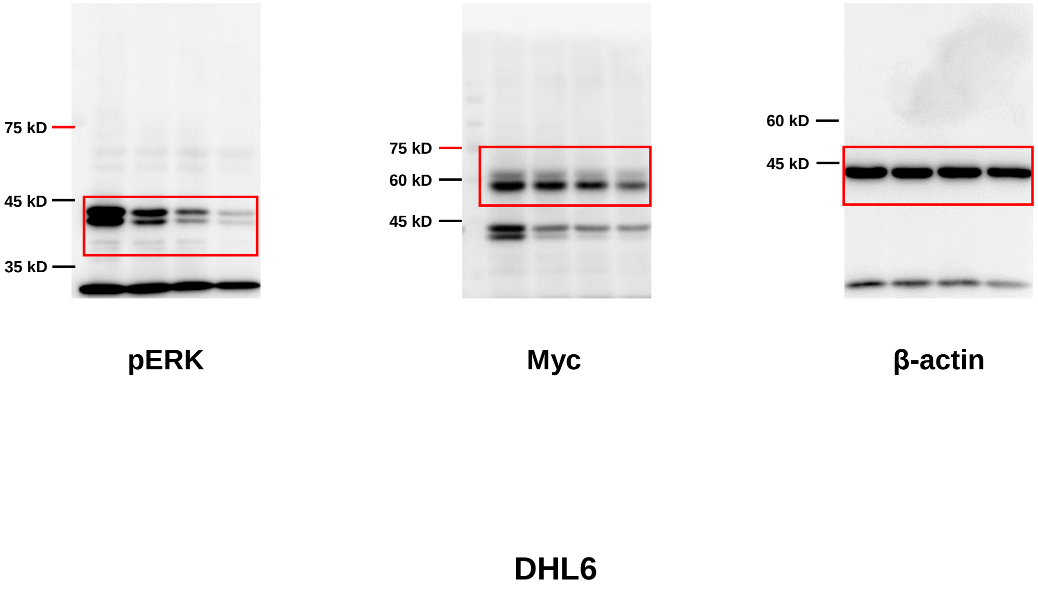

Supplementary Figure S10A. Full size blots of Figure6B - Ly1

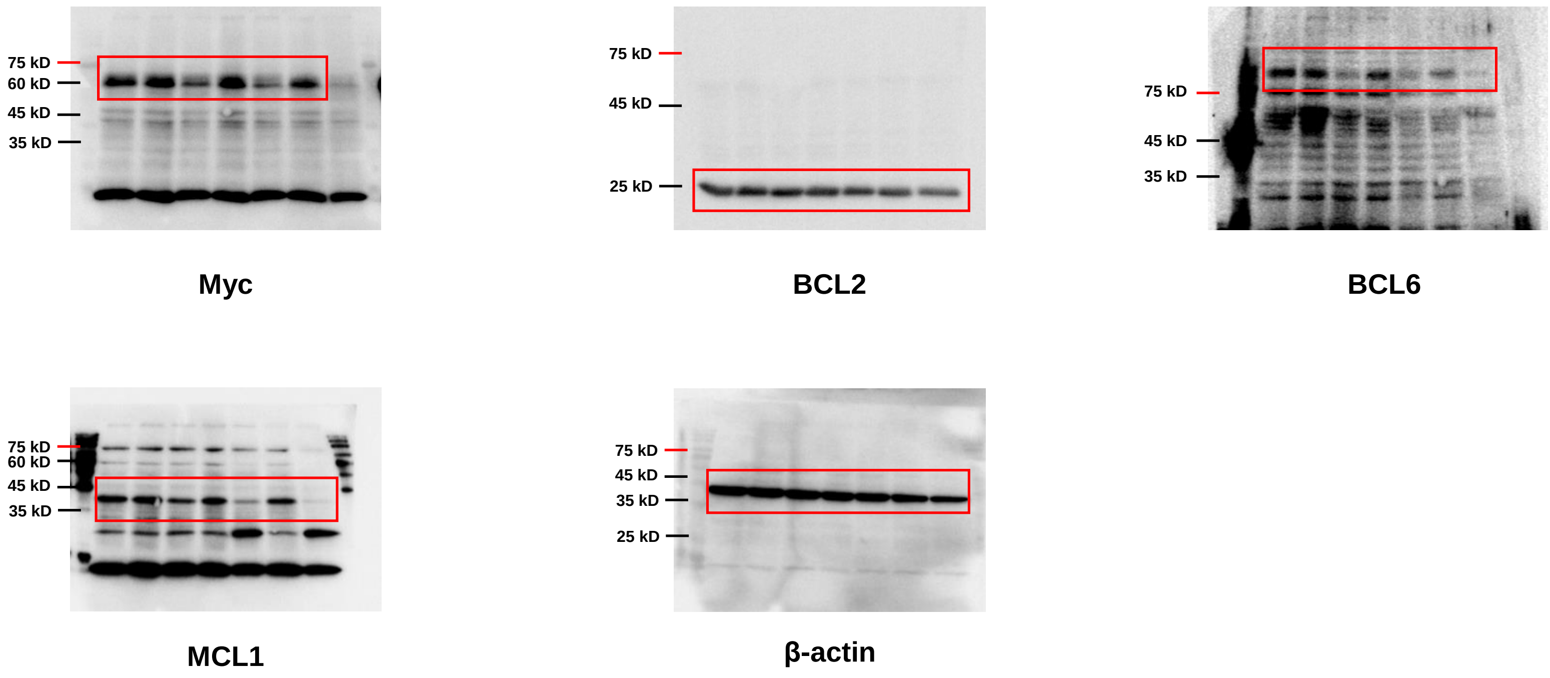

Ly1

Supplementary Figure S10B. Full size blots of Figure6B - Ly10

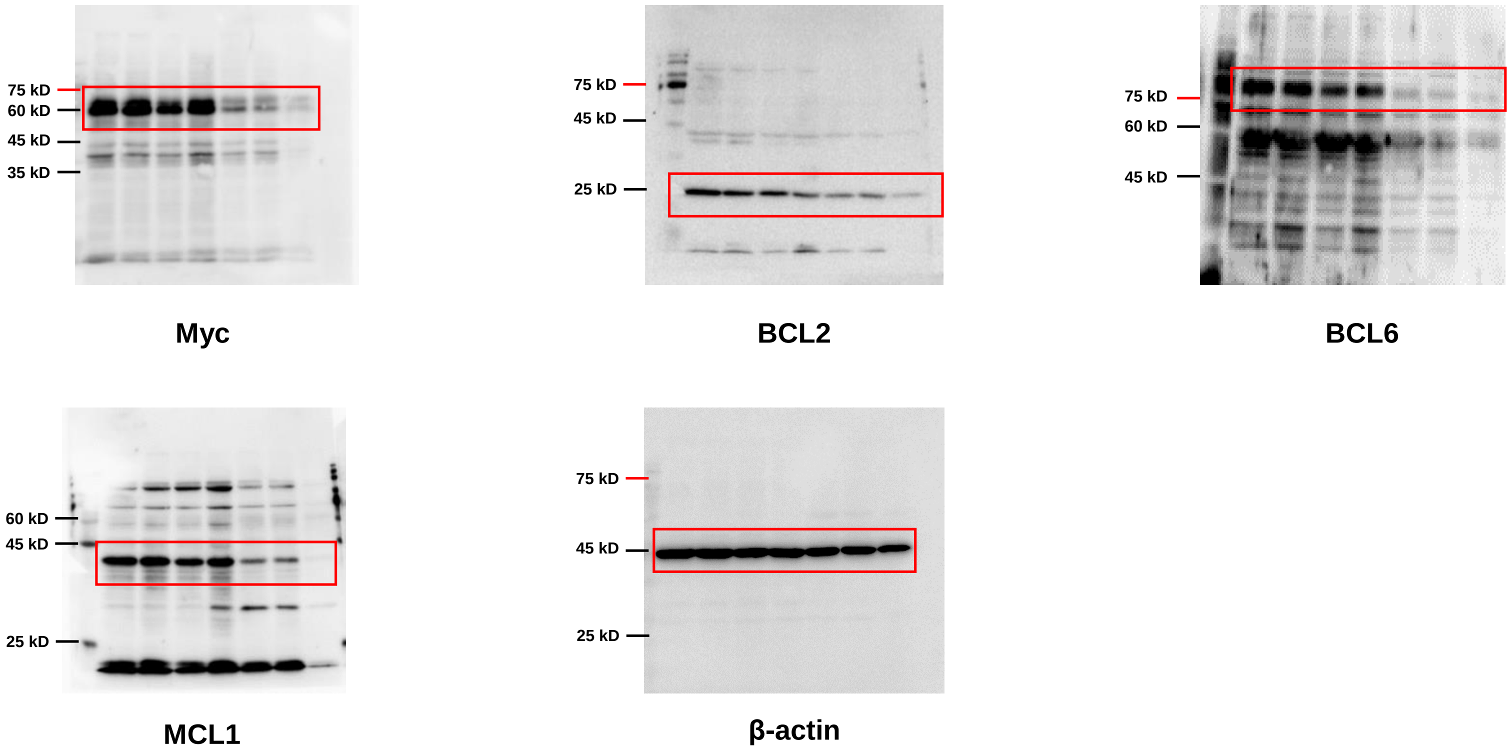

Ly10
